# Neuroinflammation driven by human immunodeficiency virus-1 (HIV-1) directs the expression of long noncoding RNA RP11-677M14.2 resulting in dysregulation of neurogranin in vivo and in vitro

**DOI:** 10.1186/s12974-024-03102-x

**Published:** 2024-04-24

**Authors:** Roberta S. dos Reis, Marc C. E. Wagner, Savannah McKenna, Velpandi Ayyavoo

**Affiliations:** Department of Infectious Diseases and Microbiology, School of Public Health, University of Pittsburgh, 2117 Pitt Public Health, 130 DeSoto Street, Pittsburgh, PA 15260 USA

## Abstract

Neuroinflammation and synaptodendritic damage represent the pathological hallmarks of HIV-1 associated cognitive disorders (HAND). The post-synaptic protein neurogranin (Nrgn) is significantly reduced in the frontal cortex of postmortem brains from people with HIV (PWH) and it is associated with inflammatory factors released by infected microglia/macrophages. However, the mechanism involved in synaptic loss have yet to be elucidated. In this study, we characterized a newly identified long non-coding RNA (lncRNA) transcript (RP11-677M14.2), which is antisense to the NRGN locus and is highly expressed in the frontal cortex of HIV-1 individuals. Further analysis indicates an inverse correlation between the expression of RP11-677M14.2 RNA and Nrgn mRNA. Additionally, the Nrgn-lncRNA axis is dysregulated in neurons exposed to HIV-1 infected microglia conditioned medium enriched with IL-1β. Moreover, in vitro overexpression of this lncRNA impacts Nrgn expression at both mRNA and protein levels. Finally, we modeled the Nrgn-lncRNA dysregulation within an HIV-1-induced inflammatory environment using brain organoids, thereby corroborating our in vivo and in vitro findings. Together, our study implicates a plausible role for lncRNA RP11-677M14.2 in modulating Nrgn expression that might serve as the mechanistic link between Nrgn loss and cognitive dysfunction in HAND, thus shedding new light on the mechanisms underlying synaptodendritic damage.

**Supplementary Information:**

The online version contains supplementary material available at 10.1186/s12974-024-03102-x.

## Introduction

HIV-1 associated neurocognitive disorder (HAND) remains as one of the most prevalent HIV-1 related comorbidities, despite systemic viral suppression [1]. The infectious microenvironment established upon viral entry in brain provokes changes in neuronal structure and function, as dendritic simplification and synaptic loss, particularly in the frontal cortex [2, 3]. These structural and functional changes are paired with deficits in memory and cognition, increasing the risk of poorer health outcomes in people with HIV-1 (PWH). Currently, there is no treatment that can prevent or restore such damage. Therefore, it is critically important to understand the molecular and cellular mechanisms behind neuronal dysregulation and cognitive impairment upon HIV-1 infection of the human brain.

We have previously reported that Neurogranin (Nrgn), a post-synaptic protein present in dendrites which is directly associated with cognitive processes such as learning, memory and executive functions, is dysregulated in the brain of PWH [4, 5]. Consequently, loss of Nrgn contributes to establishment of the synaptodendritic damage, the neuropathological hallmark underlying the cognitive deficits observed in HAND [4, 6]. However, very little is known regarding Nrgn expression, and the mechanism(s) regulating HIV-1 induced Nrgn dysregulation.

Non-coding RNAs (ncRNAs) contribute to gene expression through modulation of transcriptional and post-transcriptional processes [7–9]. The long non coding RNAs (lncRNAs) can bind to several proteins, DNAs and RNAs to form functional complexes to regulate gene expression from epigenetic modifications, transcription to RNA processing, transport and translation [9, 10]. In recent years, thousands of lncRNAs have been annotated in human genome and the number of reports suggesting their functionality and implications to physiological and pathological processes are rapidly increasing. Therefore, a growing number of lncRNAs have also been reported to be altered in response to viral infections [5, 11–14]. Nevertheless, only few long ncRNAs have been functionally characterized to date and have been found to be manipulated by HIV-1 [15–17].

In this study we have identified a lncRNA transcript (RP11-677M14.2) localized in the antisense strand in NRGN locus that has not been functionally characterized. A number of natural antisense transcripts have been recently investigated and characterized as regulators of their sense mRNA expression [18–21]. Moreover, compared to intergenic and intronic localized lncRNAs, antisense lncRNAs are more stable [22], which might be good indicators for their potential biological functions. Therefore, we hypothesize that RP11-677M14.2 antisense lncRNA might regulate Nrgn mRNA expression in neurons.

Here, we report the characterization of the RP11-677M14.2 antisense lncRNA, with a focus on its physiological relevance to Nrgn and HIV-1 neuropathology. We found that RP11-677M14.2 transcript is significantly enriched in frontal cortex tissue of PWH. Interestingly, our dada showed that higher RP11-677M14.2 expression in frontal cortex is directly correlated with lower Nrgn mRNA expression. Accordingly, overexpression of this lncRNA in neurons resulted in reduced Nrgn expression through mechanisms other than physically interacting with Nrgn mRNA. Furthermore, two-dimensional, or three-dimensional neuronal culture treatment with conditioned media from HIV-infected macrophages/microglia induced the upregulation of the lncRNA and subsequent downregulation of Nrgn mRNA. Based on these observations, we identified a new sense and antisense regulatory axis that might represent an important missing link between inflammation and synaptic dysregulation in HIV-1 neuropathogenesis.

## Methods

### Study cohort

Tissue samples (frontal cortex) from people with HIV-1 with and without cognitive impairment were obtained from National NeuroAIDS Tissue Consortium (NNTC) and Multicenter AIDS Cohort study (MACS). Frontal cortex samples from people without HIV-1 (HIV-1 seronegative), neurocognitive normal individuals were obtained from NIH Neurobiobank (University of Miami Brain Endowment Brank) and used as control. To eliminate age and sex as cofounders in this study, we used age- and sex-matched (all male) study population. Diagnosis of HAND was based on the clinical classification redefined in 2007 [23]. The use of postmortem tissue was reviewed and approved by Committee for Oversight of Research and Clinical Training Involving Decedents (CORID) of the University of Pittsburgh. Summary of characteristics and demographics information is listed in Supplementary Table 1, Additional file 2. All frozen tissue samples were preserved frozen at − 80 °C until required. FFPE slides were stored at room temperature until required. Blood samples from healthy adults PBMCs were obtained from Red Cross Blood bank.

### Cells

HEK293T and U87MG (NIH AIDS Reagent program), SH-SY5Y (ATCC) cells were grown in DMEM supplemented with 10% FCS, 1% glutamine and 1% penicillin-streptomycin. We induced differentiation of SH-SY5Y cells by adding 10 mM all‐trans‐retinoic acid (RA) to the growth medium 24 h post seeding, and RA‐containing growth medium was replaced every day for 7 consecutive days. Primary adult human microglia were a gift from Dr. Changiz Geula from Northwestern University. Briefly, microglia were isolated from the prefrontal cortex of a 71-year-old Caucasian male (*postmortem* interval of 31 h). Brain tissue from this patient was obtained from Northwestern University Alzheimer’s Disease Center Brain Bank (AG13854). Culture was maintained as previously published [24]. Briefly, cells were seeded in PDL-coated plates and kept in complete microglia medium (ScienCell Research Laboratories). Experiments were conducted with passages between 8 and 10. All cell lines were kept in a humidified incubator at 37 °C and 5% CO_2_.

### Isolation of CD14 + monocytes and differentiation to macrophages (MDM)

Monocytes-derived macrophages (MDMs) were generated from normal peripheral blood mononuclear cells (PBMC). PBMCs from healthy donor were isolated by Ficoll-Hypaque gradient centrifugation. CD14 + monocytes were purified by positive selection using anti-CD14 monoclonal antibody-coated magnetic microbeads (Miltenyi Biotech, Auburn, CA) and differentiated in presence of 1 pg/ml M-CSF and 1 × 10^6^ IU/ml GM-CSF (R&D Systems) as described previously [25]. Half the volume of media was replaced every third day with fresh media containing GM-CSF and M-CSF for 7–8 days to differentiate them into MDMs.

### Virus production

HIV-1 viruses were generated using the neurotropic proviral construct pNL43-YU2 Env-EGFP. HEK293T cells (2 × 10^6^) were transfected with 3.5 mg of proviral construct and 1.5 mg of vesicular stomatitis virus G (VSV-G) -Envelope expression plasmid using 15 μL PolyJet™ transfection reagent (SignaGen Laboratories). The transfection mixture was gently vortexed and incubated for 20 min at room temperature to allow the formation of transfection complexes. The transfection mixture was then added dropwise to the cells and incubated at 37 °C for 16 h. The medium-containing transfection mixture was replaced using fresh complete medium, and after another 48 h the supernatant containing viruses was removed, spun at 3000 g for 10 min and filtered to remove cell debris. Viruses were collected by ultracentrifugation for 60 min at 20,000 rpm (4˚C) and stored at -80˚C until further use. Viruses were titrated using U87MG CD4 + CCR5 + permissive cells to determine the infectivity as infectious units/ml. Adult primary microglia and MDMs were infected with HIV-1 at a multiplicity of infection of 0.5 as described before [4]. Mock infection was performed using equal amount of HEK293T supernatant. Microglia supernatants were collected 3 to 14 days post infection and MDM supernatants were collected 8 to 12 days post infection [4]. Supernatants were treated with complete protease inhibitors (Roche) and stored at -80 °C until use.

### Plasmids construction and gene delivery

The cDNA encoding full length RP11-677M14.2 was PCR-amplified from undifferentiated SH-SY5Y (ASNrgnHindIII_Fw 5’- CGAAGCTTCTTTCAGTACCAGGATTCTTTGGG-3’; ASNrgnXhoI_Rev 5’-CGCTCGAGAGGTACGTAATAGCTTTATTTTGGGG-3’) and subcloned into pcDNA3.1 vector (Invitrogen). The empty pcDNA3.1 vector was used as the control. All plasmids were isolated using GeneJET Plasmid Maxiprep kit (ThermoFisher) and the specific clones were confirmed by DNA sequencing of at least 5 colonies. HEK293T and SH-SY5Y (500.000 cells) were transfected with 1 µg of proviral construct using 3 µL PolyJet™ transfection reagent (SignaGen Laboratories) per manufacturer’s instructions. For lentiviral transduction, 3 short harpin RNAs (shRNAs) targeting different sequences in RP11-77M14.2 were cloned into pLV[shRNA]-EGFP mammalian shRNA knockdown lentiviral vector (customized by Vector Builder). VSV-G-Env pseudotyped lentiviral particles were produced by transient transfection of HEK293T cells using ViraPower™ lentiviral packaging mix (Invitrogen) per manufacturer’s instructions. Both HEK293T and SH-SY5Y were transduced with infectious lentiviruses in the presence of polybrene (Sigma) diluted at 5 µg/mL of medium. The assays were conducted 24–48 h after transfection and 72 h after transduction.

### Northern blot analysis

RP11-677M14.2 transcript size and splicing variants were confirmed by northern blotting using the HCR- Northern Blot technology (Molecular Instruments). A total of 10 µg of RNA was electrophoresed on a 1% denaturing agarose gel and was transferred to a positively charged nylon membrane by capillary transfer. The RNA was then fixed to the membrane using UV crosslinking. The cross-linked membrane was prehybridized with hybridization buffer and hybridized with multiple RP11-677M14.2-specific oligonucleotide HCR-probes at 37 °C in roller bottles overnight. Signal amplification was carried out in amplification buffer in roller bottles for 4 h at 37 °C. The multiple RP11-677M14.2-specific probes were designed by Molecular Instruments according to the RNA sequence.

### Total RNA extraction and quantitative real time PCR

RNA was isolated from tissue and cells using the MirVana kit (ThermoFisher) per manufacturer’s recommendations. The concentration and purity of the RNA were measured by a NanoDrop 1000 spectrophotometer (Thermo Fisher Scientific). Purity was checked by the ratio of the OD_260_/OD_280_ and OD_260_/OD_230_. The RNA was treated with DNase using a DNA-free Turbo DNase kit (Ambion). cDNA was prepared from 1 µg of total RNA using a high-capacity cDNA reverse transcription kit (ThermoFisher) in 20 µL total volume reaction. Quantitative real time PCR was performed using Taqman Universal PCR master mix (ThermoFisher) and the appropriate Taqman assays (ThermoFisher) or primers (Supplementary Table 2, Additional file 2) with 2 µL of the RT reaction mixture. Assays were conducted on Thermo ABI ViiA7 real time PCR system in the following cycling conditions: activation of Taq DNA polymerase at 95 °C for 10 min, followed by 45 cycles of amplification at 95 °C for 15 s and 60 °C for 1 min. Results were normalized to the expression of Ribosomal Protein Lateral Stalk Subunit P0 (RPLP0) (Supplementary Table 2, Additional file 2).

### Subcellular fractionation

The separation of nuclear and cytosolic fractions was performed using a PARIS Kit (Life Technologies) according to the manufacturer’s instructions. RNA extraction of the fractions was performed as previously described in methods and compared to total cell RNA extracts using GAPDH and *Malat 1* as cytoplasmic and nuclear controls respectively (Supplementary Table 2, Additional file 2).

### RNA hybridization chain reaction - fluorescence in situ hybridization (RNA HCR-FISH)

Multiple HCR-FISH probes were designed by Molecular Instruments to hybridize to various sequences along the Nrgn mRNA and RP11-677M14.2 transcript. For simultaneous amplification in different color channels, HCR initiators were appended to tiled sequences via a 2-base spacer (AA) creating even- and odd-tiled sequences probes. For culture cells, we used 4.8 pmol of each of the pooled HCR RNA FISH probes sets per 300 µL of hybridization buffer (Molecular Instruments) per coverslip, according to manufacturer’ protocol. Hybridization of initiation probes was carried out in humidified chamber overnight at 37˚C. Before the amplification step, we snap-cooled 6 µL per well of individual 3 µM HCR hairpins amplifiers (Molecular Instruments) conjugated to Alexa Fluor 647 (Alexa 647) or Alexa Fluor 488 (Alexa 488) in separate PCR tubes by heating at 95 °C for 90 s and immediately transferring it to room temperature for 30 min protected from light. Next, we pooled the hairpins in 300 µL of amplification buffer to a final concentration of 60 nM each and added to samples after washing them with wash buffer (Molecular Instruments). The coverslips were incubated at room temperature overnight protected from light. Post hairpin amplification, we washed the samples 5 times for 5 min with 5× SSCT, added 100 µL of 100 ng/mL of DAPI (Invitrogen) solution in PBS for 1 min. Samples were mounted on slides with gelvatol and we proceeded to image them.

For detection of RNA with HCR RNA FISH on formalin-fixed paraffin-embedded (FFPR) tissues, we first deparaffinized the sections on slides with serial grades of ethanol rehydration washes. We washed the slides in nuclease-free water for 3 min, and then performed antigen retrieval by immersing the slides in a heated solution (of 10 mM sodium citrate (pH 6) for 15 min at 95 ˚C. After antigen retrieval, we rinsed the cooled slides with 1x PBS 0.1% Tween 20 and immediately proceeded to the protein detection stage, according to manufacturer’s instructions (Molecular Instruments). Briefly, sections were permeabilized with 0.1% Triton-X-PBS for 15 min followed by blocking with 2% BSA for 1 h. Next, each tissue sections was incubated with 1.6 pmol of lncRNA probe set in 100 µL of hybridization buffer overnight in the 37˚C humidified chamber. After the primary probe hybridization, we washed the samples by immersion sequentially in 75% wash buffer (Molecular Instruments) plus 25% 5× SSCT (5× SSC + 0.1% Tween 20) solution, 50% wash buffer plus 50% 5× SSCT solution, 25% wash buffer plus 75% 5× SSCT solution, and 100% 5× SSCT for 15 min each at 37 °C. We then washed the samples in 5× SSCT at room temperature for 5 min and incubated the samples at room temperature for 30 min in amplification buffer (Molecular Instruments). During this incubation, we snap-cooled, by heating at 95 °C for 90 s in separate PCR tubes, 2 µL per slide of individual 3 µM HCR hairpins (Molecular Instruments) conjugated to Alexa 647 and immediately transferred the samples to room temperature to cool for 30 min protected from light. After, we pooled the hairpins in 100 µL of amplification buffer per slide to a final concentration of 60 nM each. We added the hairpin solution to samples, placed a glass coverslip on top, and then incubated samples at room temperature overnight protected from light. After hairpin amplification, we washed samples 1 × 5 min in 5× SSCT, 2 × 15 min in 5× SSCT, and then 1 × 5 min with 5× SSCT again. We then stained nuclei by adding 100 µL of 5× SSCT containing 100 ng/mL of DAPI to each slide for 5 min at room temperature, added mounting media and coverslip, and then proceeded to image the samples.

### Generation of brain organoids and RNA extraction

To generate brain organoids incorporated with mock and HIV-1-infected microglia we followed the protocol previously established [26]. Briefly, microglia (both mock and HIV-infected) were detached from the 2D flasks and incubated with two-week old human brain organoids (hBORGs), previously rinsed with PBS, as the ratio of 1 microglia cell to 20 NPCs. Microglia and hBORGs were incubated without agitation for 24 h to allow attachment of microglia to the hBORG surface. The MG-hBORGs were then carefully transferred to a new plate with fresh differentiation media to remove unattached MGs and were maintained in culture in differentiation media for an additional 30 days. To extract RNA from MG-hBORGs, we first removed the matrigel from MG-hBORGs by treating them with cell recovery solution (Corning) for 1 h under agitation at 4˚C, followed by three washes with 1x PBS. We proceeded to RNA extraction of the pellet as previously described in this Methods section.

### Immunofluorescence staining

Deparaffinized sections from frontal cortex tissues and organoids were rehydrated by three washes of PBS and five washes of 0.5% bovine serum albumin (BSA) and circled with a Liquid Blocker Mini Pap Pen (Life Technologies). Sections were further permeabilized with 0.1% Triton-X-PBS for 15 min followed by blocking with 2% BSA for 1 h. Sections were incubated with primary antibodies against human Nrgn (1:1000-kindly donated by Drs. Everett and Yang from John Hopkins University) overnight at 4 °C. Tissues were washed five times with 0.5% BSA in PBS and were further incubated with donkey anti-rabbit-Cy3 and MAP-2-AF488 (1:200-Merck) followed by five washes with 0.5% BSA in PBS, and the nuclei were stained with DAPI (1:1000).

Coverslips from SH-SY5Y cells were fixed in 4% paraformaldehyde for 15 min, permeabilized and stained as previously described [4]. Lastly, slides were mounted with Gelvatol mounting medium and images were taken using confocal microscope.

### Confocal microscopy and image analysis

Confocal imaging was carried out using a Z-stacking function on the Nikon A1 inverted confocal microscope using 40X dry or 60X oil objective at 4X zoom. For every image, we used a step size of 0.3–0.5 μm for coverslips and paraffin sections totalizing 20–30 steps to image the entire layer of cells. Images shown are representative of cultures generated from 3 independent experiments (4 independent images/coverslip). Maximum intensity Z-projections were generated and analyzed using FIJI ImageJ 2.14/ 1.54f (National Institute of Health, USA).

### Statistical analysis

Statistical analyses were performed using GraphPad Prism v 9.5.1 for Mac OSX (GraphPad Software, La Jolla, California USA). Three independent experiments were performed for all cell biological assays, unless otherwise stated. Experimental results were presented as the mean ± SE (standard error) and the difference were evaluated using the Student’s *t*-test with Welch’s correction. The relationship between the normalized expression (delta CT values) of Nrgn mRNA and RP11-677M14.2 transcript in same brain tissues was analyzed by Pearson’s correlation. *P* < 0.05 was considered to indicate a statistically significant difference.

## Results

### HIV-1 infection decreases Neurogranin (Nrgn) expression in human brain tissue both at the mRNA and protein levels

**Fig. 1:**
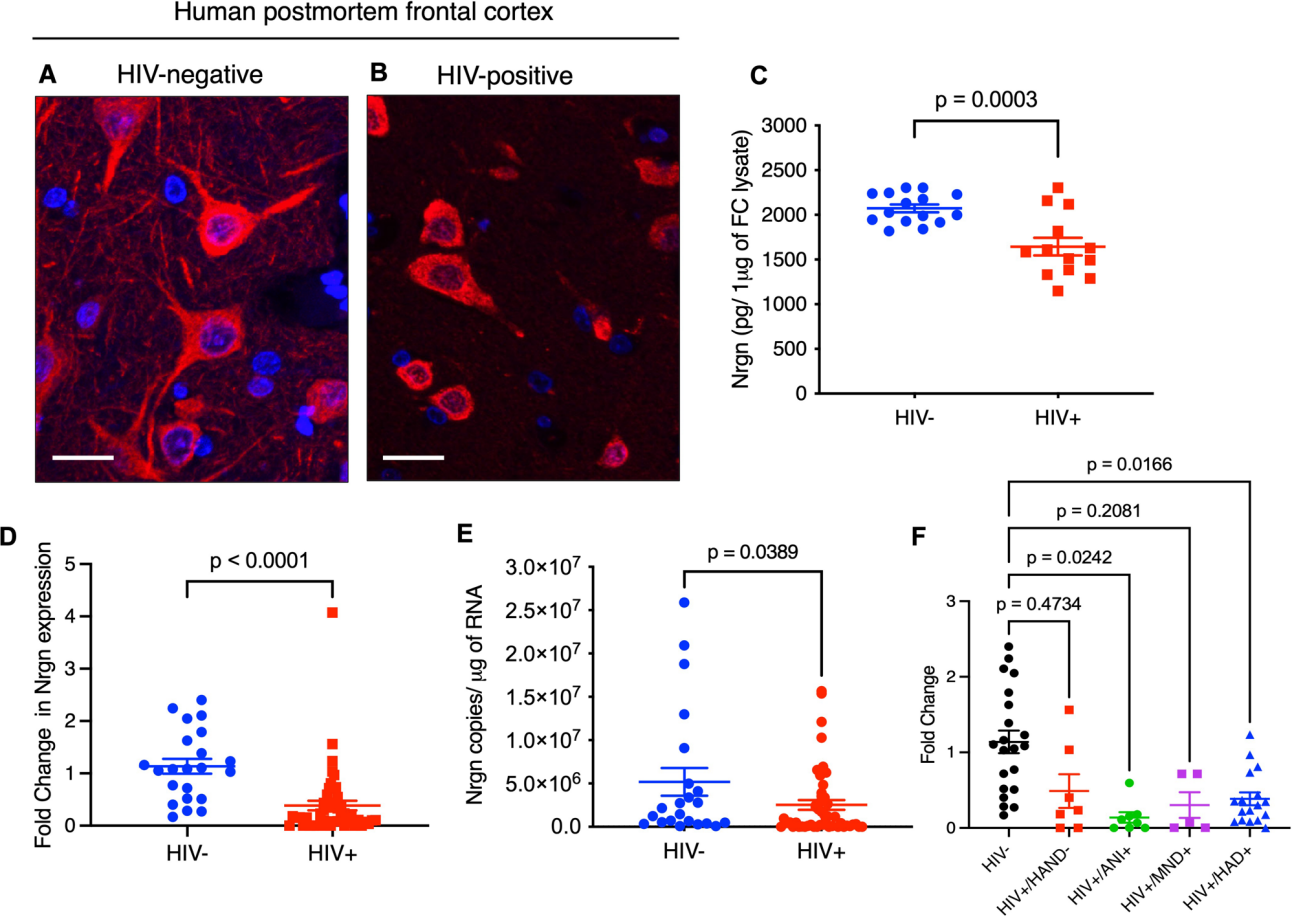
**Neurogranin (Nrgn) is dysregulated in PWH at the mRNA and protein levels.** Representative images of postmortem frontal cortex (FC) neurons stained for Nrgn (Cy3, red) and DAPI (blue) in (**A**) control and (**B**) people with HIV-1. The images are z-projections of image stacks acquired at ×60 magnification; scale bar is 10 μm. (**C**) Quantification of Nrgn levels in whole frontal cortex lysates from people with HIV-1 (*N* = 13) in comparison with control individuals (*N* = 15). (**D**) Relative expression of Nrgn mRNA in FC brain samples from HIV-1 positive individuals (*N* = 49) compared to control individuals (*N* = 22) as assessed by RT-qPCR. (**E**) Quantification of Nrgn mRNA copy number in FC samples of HIV-1 positive individuals compared to control individuals. (**F**) Comparison of Nrgn expression across different clinical stages of HAND

Simplification of dendritic network and synaptic dysfunction are neuropathological hallmarks of early HAND [3, 27, 28]. Previous report from our group indicated that post-synaptic protein, Nrgn is dysregulated in HAND positive individuals [4]. We then sought to evaluate the correlation between Nrgn expression and HIV-1 infection in the human brain cortex. Using immunofluorescence, we observed a dramatic decrease in Nrgn expression in frontal cortex tissue of HIV-positive individuals compared to healthy control brain tissues (Fig. 1A-B). We further quantitated the Nrgn level in whole frontal cortex tissue lysates in PWH (HIV-1+) (*N* = 13 donors) and age-matched people without HIV (HIV-) (*N* = 15) by Nrgn ELISA. We observed an average of 20% reduction in Nrgn level in HIV-1-positive individuals (Fig. 1C), compared to HIV-1 negative controls. Next, we assessed the relative expression level of Nrgn mRNA in total RNA samples from frontal cortex of HIV-1-positive individuals (*N* = 22) and age-matched control uninfected brain samples (*N* = 49) by RT-qPCR. Our results indicate that relative Nrgn mRNA level is significantly lower (average of 2.5-fold) in PWH in 75.5% of cases (37 out of 49) (Fig. 1D). Further assessment of copy number of Nrgn mRNA in brain tissue indicate that Nrgn is significantly reduced by 2.1-fold in HIV-1 positive individuals compared to HIV-1 negative individuals (Fig. 1E) based on the curve (Additional file 1, Fig S1A), confirming that decreased level of Nrgn protein in frontal cortex of PWH is associated with reduced amount of Nrgn mRNA. Next, we sub-classified the Nrgn expression level based on HAND status, varying from unimpaired (HAND-negative), asymptomatic neurocognitively impaired (ANI), mildly impaired (MND) to the most severe form of disease (HIV-1- associated dementia, HAD) (Fig. 1F). Intriguingly, we found that Nrgn is significantly reduced early in the disease stage, suggesting that Nrgn dysregulation occurs before the onset of the clinical symptoms of cognitive impairment. These results were further confirmed through immunohistochemistry by co-staining Nrgn and microtubule associated protein 2 (MAP-2) (Additional file 1, Fig. S1B-C). MAP-2 is one of the major components of neuronal cytoskeleton and is highly concentrated in dendrites, thus, it is considered a marker for dendritic integrity [29]. As expected, Nrgn is enriched in neurons of healthy control samples and widely distributed throughout cell body and dendrites (Additional file 1, Fig. S1B, yellow, overlay). Notably, a substantial loss of Nrgn in dendrites is observed HAND-negative PWH donors, demonstrating that HIV-1-driven Nrgn dysregulation precedes the loss of dendritic integrity.

### The expression of the candidate Nrgn regulator, lncRNA RP11-677M14.2 transcript is elevated in brain of HIV-1 positive individuals

To identify the putative lncRNAs and its potential role in neurogranin dysregulation, we utilized the University of California Santa Cruz (UCSC) Genome Browser (genome.ucsc.edu) to investigate the genomic landscape of human NRGN gene. We have identified a single transcript (RP11-677M14.2) of 1,704 bp length, which is localized in the antisense strand in NRGN locus in chromosome 11 (-strand, hg38) that remains to be characterized (Additional file 1, Fig. S2A, red arrow). Moreover, in Ensembl (http://www.ensembl.org) browser, this transcript corresponds to a 3 exons antisense RNA with no protein-coding potential, being classified as a long non-coding RNA. RP11-677M14.2 arises from independent promoter and its promoter region co-aligns to epigenetic markers of active transcription (Additional file 1, Fig. S2B, red circle, H3K27Ac mark). Analysis of the promoter activity from the same cell lines (Additional file 1. Fig. S2C, Regulatory build track, thick red blocks) corroborates to this analysis, showing that when the Nrgn promoter is inactive (grey boxes on left), the RP11-677M14.2 promoter is active (red blocks on right). Additionally, gene expression data in 53 tissues from GTEx RNA-seq track revealed that RP11-677M14.2 is particularly abundant in human brain regions where Nrgn is also abundant, such as hippocampus and frontal cortex (Additional file 1, Fig. S2D, yellow track) [30]. Together, these data suggest a potential functional role for RP11-677M14.2 in mediating Nrgn levels. RP11-77M14.2 is poorly conserved as a clear orthologous counterpart for this lncRNA could not be identified when examining its full cDNA and RNA sequences across 100 vertebrate’s species using the PhyloP and Multiz Alignments tracks in genome browser (Additional file 1, Fig. S2E, red arrow and red-highlighted sequences). Northern blot analysis and RT-qPCR confirmed that RP11-677M14.2 is constitutively expressed in human brain and cell lines, as well as the presence of one mature transcript of circa 500 bp (Additional file 1, Fig. S2F-G).

**Fig. 2:**
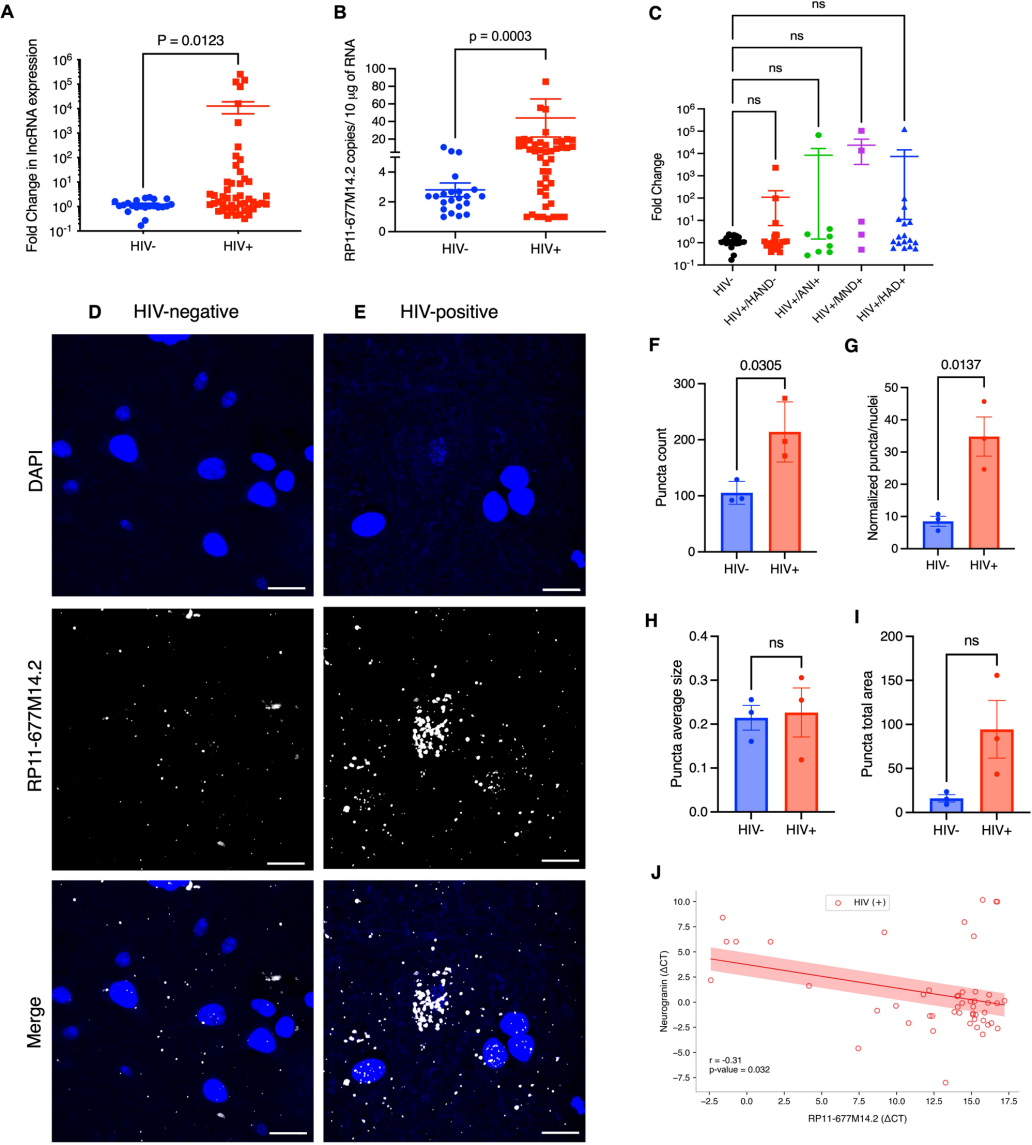
**Expression analysis of RP11-677M14.2 transcript in frontal cortex tissues.** (**A**) Relative expression of RP11-677M14.2 in FC from people with HIV-1 (*N* = 49) compared with control individuals without HIV-1 (*N* = 22) by RT-qPCR. (**B**) Quantification of RP11-677M14.2 transcript copy number in FC samples of HIV-1 positive individuals compared to control individuals. (**C**) Comparison of RP11-677M14.2 expression across different clinical stages of HAND. (**D-E**) Representative images of single molecule RNA HCR FISH with probe sets for RP11-677M14.2 transcript (AF-647, white puncta) in FC samples from people with and without HIV-1 (*N* = 3 per group). DAPI stain for cell nuclei is shown in blue. Scale bars show 10 μm. The images are z-projections of image stacks acquired at ×60 magnification. Quantification of the fluorescence signal from the experiment in panels D-E. For each the HIV-negative and HIV-positive data set, we quantified (**F**) total puncta count per image, (**G**) total puncta count normalized per cell number, (**H**) puncta average size and (**I**) puncta fluorescence signal intensity from averaged 5 images per individual per condition. (**J**) Pearson’s correlation analysis (red line) between the normalized expression of Nrgn mRNA and the normalized expression of RP11-677M14.2 in 49 HIV + frontal cortex samples (red circles) assessed by RT-qPCR. The shaded red area represents the confidence interval (*R*=-0.31, *P* = 0.032)

To investigate whether RP11-677M14.2 transcript is dysregulated in HIV-1 brains, we measured the lncRNA expression levels in frontal cortex tissues from age and sex-matched people with and without HIV by RT-qPCR assay. The level of RP11-677M14.2 was aberrantly up-regulated (> 12.00 average fold-change, *p* = 0.0123) in 61.2% (30 of 49) of PWH compared with tissues without HIV-1 (Fig. 2A). We also quantified the copy number of RP11-677M14.2 transcripts in brain tissue of PWH (HIV+) and compared with people without HIV (HIV-) (Fig. 2B) based on the curve (Additional file 1, Fig S2H). This analysis also revealed a statistically significant increase in RP11-677M14.2 numbers in PWH (*p* = 0.0003) confirming the relative quantification assessment. Moreover, the relationship between RP11-677M14.2 expression and clinical stages of HAND was analyzed. Although not statistically significant, there is an upward trend of increased RP11-677M14.2 levels as the disease progresses (Fig. 2C). To further confirm RP11-677M14.2 differential expression, next, we performed RNA-FISH HCR for RP11-677M14.2 transcript and co-stained with DAPI in 3 paired HIV-positive and HIV-negative FFPE autopsied human frontal cortex tissues. As shown in Fig. 2D and E, the lncRNA RP11-677M14.2 transcript is overexpressed in the brain tissue of PWH, and the subcellular localization was predominantly in the cytoplasm. Calculation of total puncta counts (Fig. 2F) and the normalized puncta counts (Fig. 2G) further confirm that this lncRNA is globally upregulated in HIV-1 positive brains. Although no significant difference in puncta size and total area was observed between the two groups (Fig. 2H and I, respectively), we observed an increased lncRNA RP11-677M14.2 puncta area in brains of PWH (Fig. 2I). Further, to investigate whether a linear relationship exists between lncRNA RP11-677M14.2 transcript and Nrgn mRNA levels, we performed Pearson’s correlation analysis in the same brain tissue samples. As depicted in Fig. 2J, we observed a statistically significant inverse correlation between delta-CT values with a correlation coefficient of -0.3065 (*p* = 0.0322), supporting the notion that the high expression of lncRNA RP11-677M14.2 correlated with lower levels of Nrgn in frontal cortex. Thus, we refer RP11-677M14.2 transcript as Nrgn antisense (Nrgn-AS).

### LncRNA RP11-677M14.2 displays both nuclear and cytoplasmic distribution

**Fig. 3:**
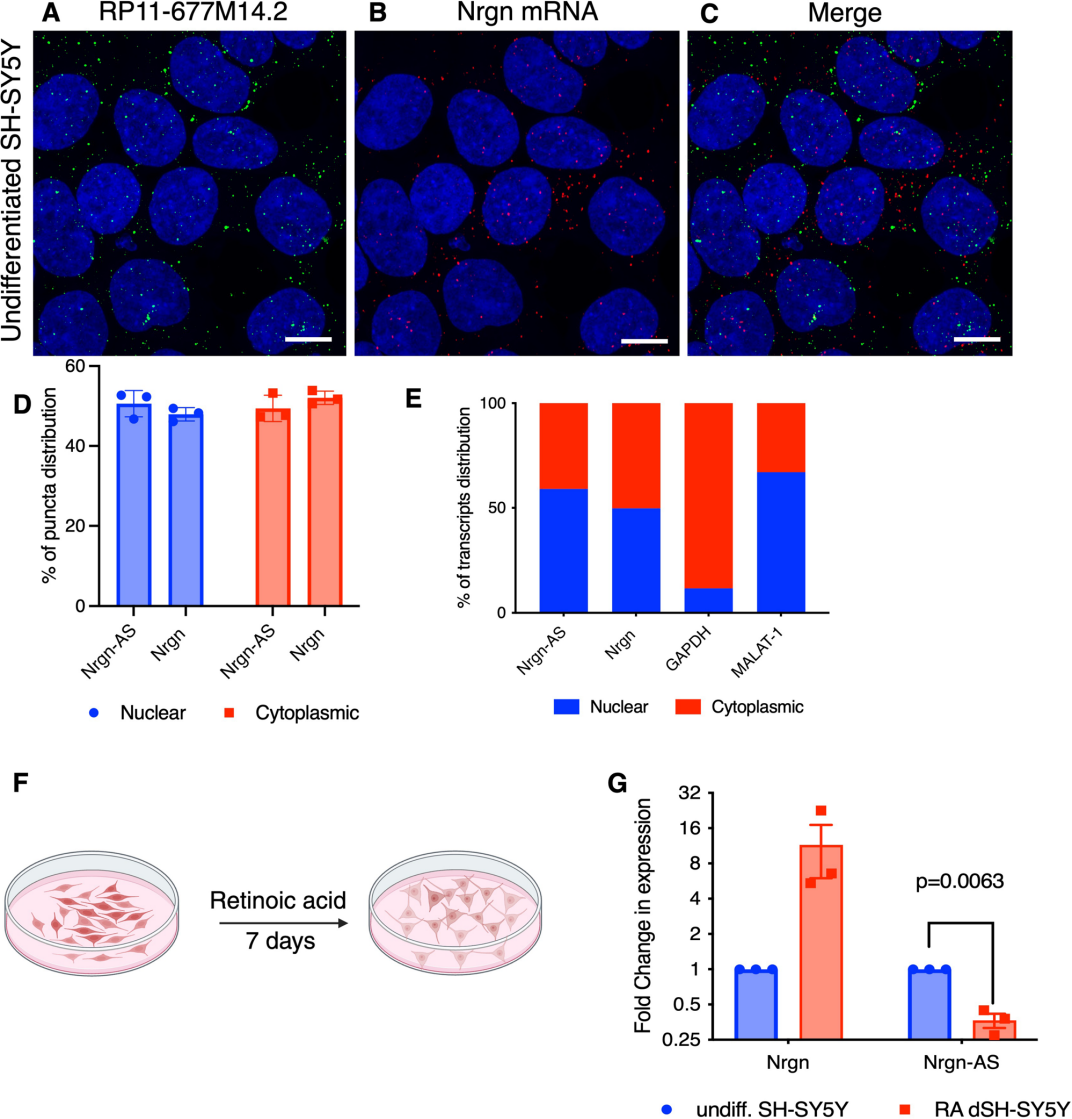
**Transcriptional activation of Nrgn is associated with repression of RP11-677M14.2 lncRNA.** Representative images of single molecule RNA multiplex HCR FISH with probe sets for (**A**) RP11-677M14.2 transcript (AF-488, green puncta) and (**B**) Nrgn mRNA (AF-647, red puncta) and (**C**) the colocalization of both transcripts in undifferentiated SH- SY5Y neuroblastoma cells. DAPI stain for cell nuclei is shown in blue. Scale bars show 10 μm, *N* = 3. The images are z-projections of image stacks acquired at ×60 magnification. (**D**) Distribution of RP11-677M14.2 transcript and Nrgn mRNA were quantified in different cellular compartments and presented as average percentage rate of total RNA puncta (*N* = 3). (**E**) Relative RP11-677M14.2 and Nrgn mRNA levels in cytoplasm or nucleus of SH-SY5Y were detected by RT-qPCR. GAPDH was used as cytoplasm control and Malat1 was used as nucleus control. (**F**) Schematic of SH-SY5Y differentiation with retinoic acid (RA) treatment. (**G**) Assessment of Nrgn mRNA and RP11-677M14.2 transcript through RT-qPCR before and after SH-SY5Y differentiation treatment. *N* = 3 independent experiments

LncRNAs have been separated into several broad classes in terms of their mechanisms of regulation of mRNA transcription and translation: decoys, regulators of translation, enhancers and modular scaffolds that guide chromatin modifying enzymes to specific genomic loci [31, 32]. Those lncRNAs localized within the nucleus, have been previously linked to the epigenetic control of transcriptional regulation through different mechanisms, whereas cytoplasmic lncRNAs are involved essentially in post-transcriptional mechanisms, subcellular localization and regulation of translation [33]. To dissect the function of Nrgn-AS in Nrgn regulation we first examined the distribution of this lncRNA (Fig. 3A) and Nrgn mRNA (Fig. 3B) in SH-SY5Y cells through RNA-FISH HCR co-stained with DAPI (Fig. 3C). Calculation of the puncta revealed that nearly 51% of the lncRNA transcripts reside in the nuclear compartment, whereas 48% of the Nrgn mRNA transcripts are localized within the nucleus (Fig. 3D). Cell fractionation followed by RT-qPCR further confirmed that both RP11-677M14.2 transcript Nrgn mRNA are equally distributed between these two subcellular compartments (Fig. 3E). According to the distribution of glyceraldehyde-3-phosphate dehydrogenase (GAPDH) and Malat1, a lncRNA enriched in nucleus, the nucleus/cytoplasm separation was successful (Fig. 3E). Notably, minimal colocalization between the two transcripts was observed, suggesting that these transcripts do not physically interact (Fig. 3C).

### Nrgn and lncRNA RP11-677M14.2 (Nrgn-AS) transcripts exhibit discordant expression pattern

Antisense transcripts may regulate expression of its sense gene at the transcriptional level (via transcriptional interference) and/or at the post-transcriptional level [7, 8, 34]. To investigate the potential regulation of Nrgn by endogenous levels of RP11-677M14.2, we treated SH-SY5Y cells with all-trans retinoic acid (RA) for 7 days (Fig. 3F) and measured the Nrgn and RP11-677M14.2 transcript by RT-qPCR. These cells express low levels of endogenous RP11-677M14.2 and high levels of Nrgn under normal conditions. During the differentiation process with RA treatment, both Nrgn mRNA and protein increase (Fig. 3G). Interestingly, our data reveal that while the expression of Nrgn increased, RP11-677M14.2 level sharply declined by 3-fold upon differentiation (Fig. 3B). This observation suggests that Nrgn level may be regulated by its anti-sense lncRNA RP11-677M14.2 in a discordant manner upon certain stimuli, as shown for other sense-antisense pairs [19, 35]. Therefore, we speculate whether this antisense lncRNA exerts a silencing effect on the Nrgn mRNA or corresponding protein abundance.

### Overexpression of Nrgn-AS, RP11-677M14.2 inhibits Neurogranin expression

**Fig. 4:**
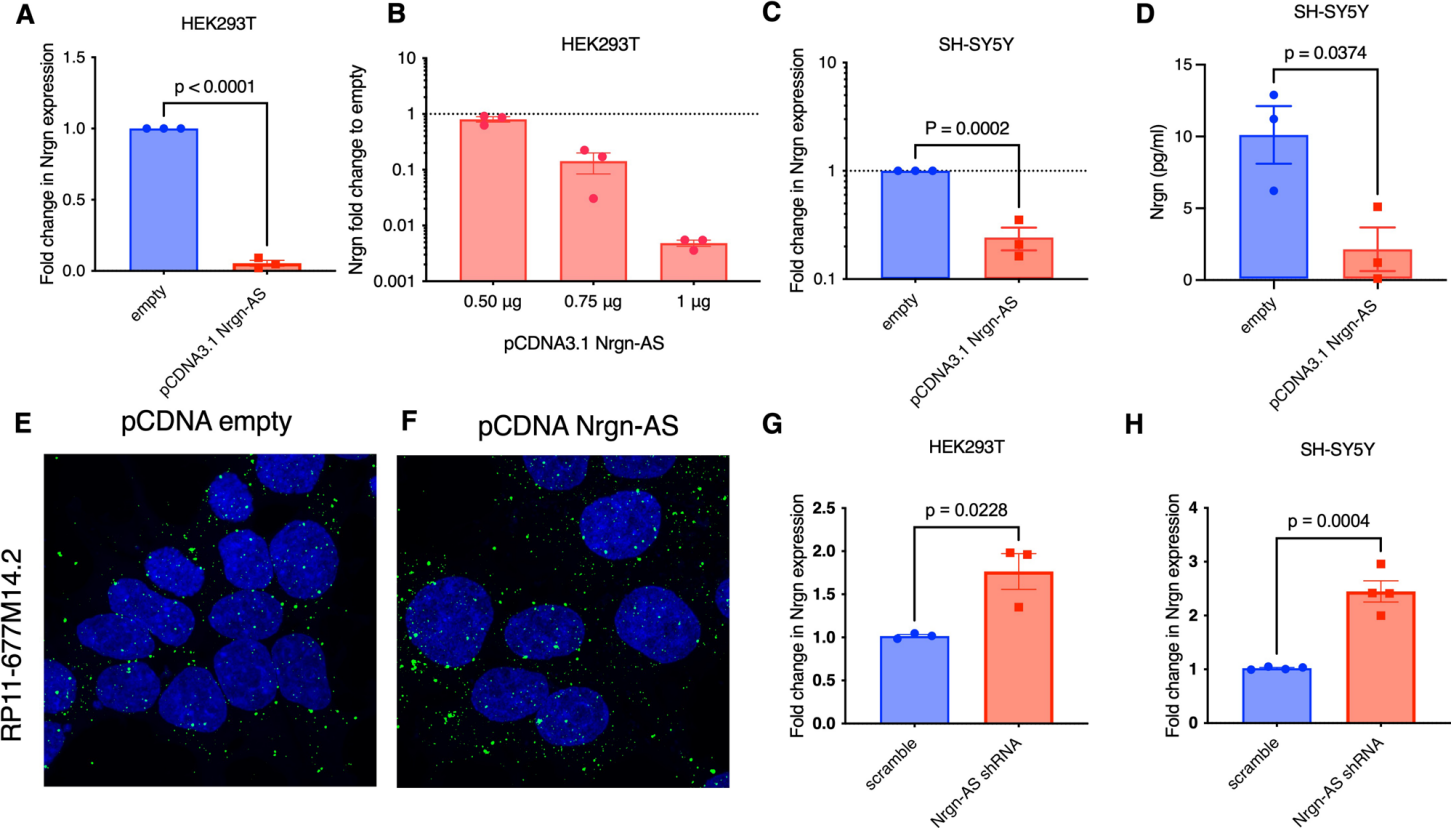
**Overexpression of RP11-677M14.2 suppresses Nrgn expression.** (**A**) HEK293T cells were transiently transfected with pCDNA3.1 RP11-677M14.2 (pCDNA 3.1 Nrgn-AS) and after 48 h the levels of Nrgn mRNA were assessed by RT-qPCR in comparison with cells transfected with empty vector. (**B**) HEK293T cells were transfected with different doses of pCDNA3.1 Nrgn-AS as indicated, and levels of Nrgn mRNA were assessed after 48 h by RT-qPCR and compared with empty vector. Levels of (**C**) Nrgn mRNA and (**D**) protein were assessed by RT-qPCR and Nrgn ELISA respectively upon stably transfection of SH-SY5Y with pCDNA3.1 Nrgn-AS compared to empty plasmid (*N* = 3). (**E-F**) Representative images of single molecule RNA HCR FISH with probe for RP11-677M14.2 transcript (AF-488, green puncta) in undifferentiated stably transduced SH-SY5Y cells compared to SH-SY5Y transduced with empty vector. DAPI stain for cell nuclei is shown in blue. Scale bars show 10 μm, *N* = 3. Effect of RP11-677M14.2 knockdown on Nrgn expression in (**G**) HEK293T and (**H**) SH-SY5Y cells assessed by RT-qPCR 72 h after transduction (*N* = 4)

To test the prediction of a discordant regulation of Nrgn-AS and Nrgn mRNA, we have cloned full-length RP11-677M14.2 transcript into pCDNA3.1 expression vector to overexpress this lncRNA in HEK293T and SH-SY5Y cells (Additional file 1, Fig. S3A). Firstly, Nrgn mRNA was assessed by RT-qPCR upon transiently transfecting the Nrgn-AS construct in HEK293T. Our results show that Nrgn mRNA was decreased by 90% in HEK293T cells in comparison to cells transfected with the empty vector (Fig. 4A). The effect seemed to be dose dependent as we observed concentration dependent decrease in Nrgn mRNA expression as we increase the quantity of pCDNA3.1 RP11-677M14.2 in transfection (Fig. 4B).

Next, we stably transfected SH-SY5Y cells with RP11-677M14.2 plasmid and observed a 50% reduction in Nrgn mRNA in these neuronal cells (Fig. 4C). To confirm these mRNA results, we next assessed Nrgn at the protein level by ELISA (Fig. 4D). Similar results were obtained when we measured the protein level of Nrgn, being observed an average decrease of 4.7-fold in neuronal cells overexpressing Nrgn-AS in comparison to cells transfected with empty vector as control. By employing FISH HCR co-stained with DAPI, we observed a diffuse distribution of the lncRNA in the stably transfected SHSY-5Y cells (Fig. 4F) similar to the endogenous distribution of this transcript observed in the empty vector control (Fig. 4E). These results indicate that the expression of Nrgn-AS directly or indirectly alters Nrgn mRNA expression resulting in lower protein levels. To investigate whether the lack of Nrgn-AS could impact Nrgn mRNA expression, we depleted Nrgn-AS expression in both HEK293T and SH-SY5Y cells through lentivirus carrying RP11-77M14.2 short-hairpin RNA (shRNA) (Fig. 4G and H, respectively). Real-time PCR displayed that knockdown of Nrgn-AS partially restored the level of Nrgn mRNA in both cell lines (Additional file 1, Fig. S3C-D).

To further investigate the effects of Nrgn-AS on the synaptodendritic damage, we also examined the expression level of selected synaptodendritic integrity markers: the dendritic marker MAP-2, the pre-synaptic markers GAP43, Synapsin and SNAP25, and the post-synaptic proteins Calmodulin, CAMK2, and calcineurin (PP3CA) in differentiated stably transfected SH-SY5Y (Additional file 1, Fig. S3B). We observed a significant decreased expression of MAP-2 (*p* = 0.016377), SNAP25 (*p* = 0.0128) and CAMK2 (*p* = 0.003930) suggesting that inhibition of Nrgn expression induced by overexpression of the lncRNA caused disruption of synaptodendritic integrity, which is implicated in cognitive decline.

### Supernatant from HIV-1 infected macrophages or microglia alters Nrgn-AS expression in neurons

**Fig. 5:**
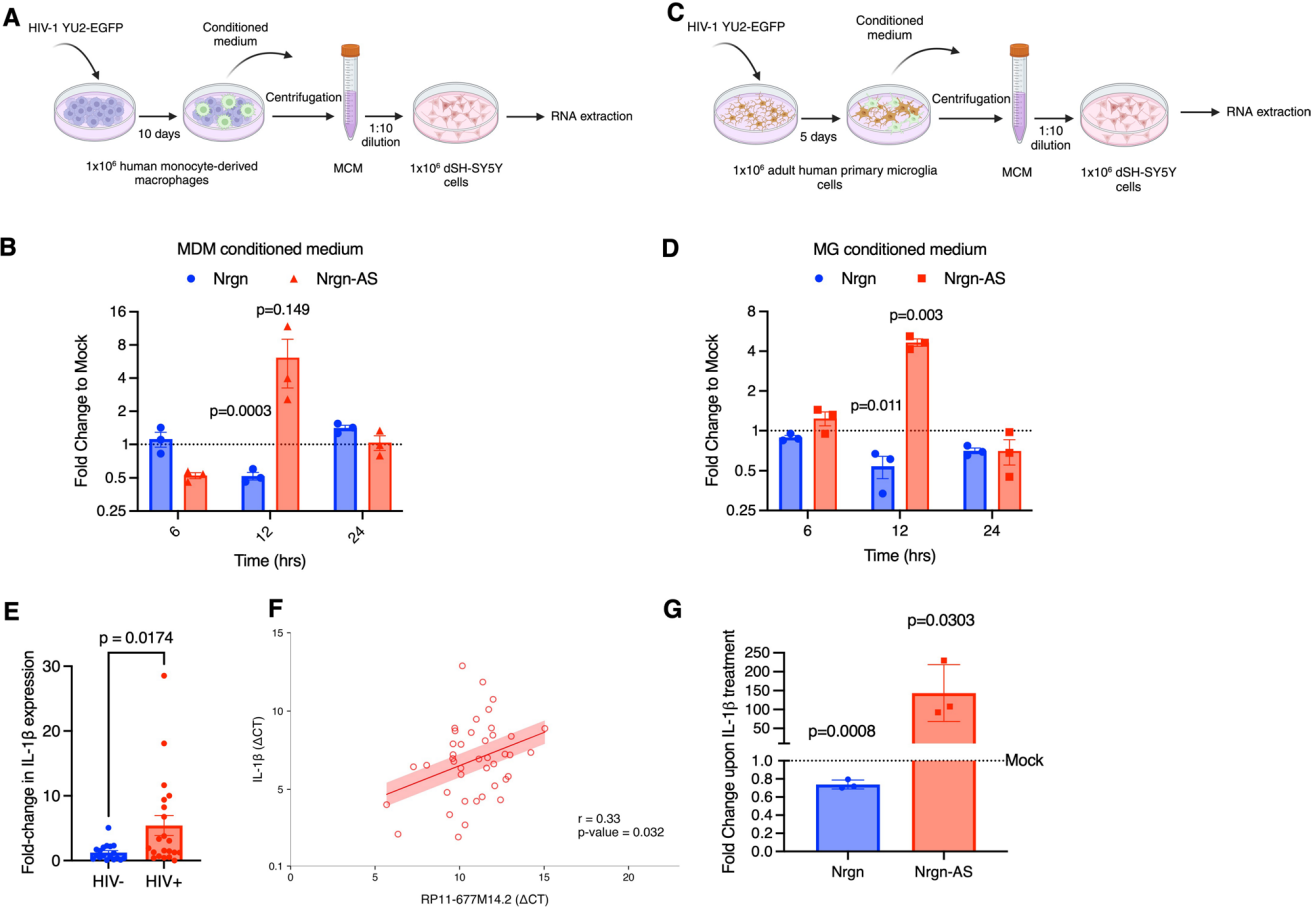
**HIV-1 infection dysregulates Nrgn-lncRNA axis in vitro.** **(A-D)** Schematic depicts experimental designed in which Nrgn-lncRNA expression axis was investigated: SH-SY5Y cells were differentiated with RA for 7 days and exposed to supernatant from HIV-1-infected or mock-infected (**A-B**) MDM or (**C-D**) Microglia. RNA was harvested at, 6, 12 and 24 h of incubation and both the RP11-677M14.2 transcript and Nrgn mRNA levels were assessed by RT-PCR. (**E**) Relative expression of IL-1β in FC brain samples from HIV-1 positive individuals (*N* = 21) compared to control individuals (*N* = 18) assessed by RT-qPCR. (**F**) Pearson’s correlation analysis (red line) between the normalized expression of IL-1β and the normalized expression of RP11-677M14.2 in 49 HIV + frontal cortex samples (red circles) assessed by RT-qPCR. The shaded red area represents the confidence interval (*R* = 0.33, *P* = 0.032) (**G**) SH-SY5Y cells were differentiated with RA for 7 days and exposed to 1 ng of recombinant IL-1β for 1 h and RNA was harvested. Nrgn and RP11-677M14.2 transcript expression levels were assessed and compared to mock-treated neurons through RT-qPCR. Dotted lines represent the mock-treated neurons expression, blue bars represent average Nrgn fold change in expression whereas red bars represent the average lncRNA fold change in expression

We next investigated whether HIV-1 infection affects Nrgn-AS transcript level as it affects Nrgn mRNA level in vitro by exposing differentiated SH-SY5Y cells to conditioned medium from HIV-infected monocytes-derived macrophages (MDM) (Fig. 5A-B), microglia cells (Fig. 5C-D) or mock-infected, to mimic the impact of HIV-1 induced inflammatory factors, toxins and/or viral proteins as previously described [36–38]. The effect of these factors on sense and antisense transcripts levels was assessed by RT-qPCR. Results indicate that exposure of cells to supernatant of HIV-infected MDMs resulted in an average 1.8-fold decrease in Nrgn mRNA and a 6.8-fold increase of Nrgn-AS levels (Fig. 5B), although the increase in the lncRNA expression varied between monocytes donors, it is not statistically significant (*p* = 0.149). Interestingly, our results show that there is a pattern of both transcripts being altered at the same time (between 6 and 12 h post-exposure) and these alterations seem to be reversible as the stress factors degrade in the culture media (∼24 h post-exposure). Similarly, we tested the conditioned media obtained from HIV-infected human microglia and observed that exposure of cells to supernatant of HIV-infected microglia resulted in a 1.86-fold decrease in Nrgn mRNA and a 4.6-fold increase in Nrgn-AS levels (Fig. 5D). Together, these results suggest that both viral proteins and/or inflammatory factors released by infected MDM or microglia affect antisense transcript level.

The composition of products released from infected MDM or microglia is complex and not fully known, however previous studies by us and others have identified several cytokines/chemokines that are known to have a role in neuropathogenesis [25, 36, 37]. Thus, to identify the proinflammatory cytokines present in HIV-1 infected MDM and microglia supernatants that might contribute to loss of Nrgn in neurons, we selected and measured the levels of interleukin (IL)-1β, IL-6, tumor necrosis factor (TNF)-α, and IL-8 in the supernatants of HIV-1 and mock-infected MDM (Additional file 1, Fig. S4A) and microglia (Additional file 1, Fig. S4B) by ELISA. As expected, HIV-1 infection increased the production and release of proinflammatory cytokines in both cell types. Among the pro-inflammatory cytokines tested, only IL-1β significantly increased upon HIV-1 infection in both MDM (*p* = 0.0210) and microglia (*p* = 0.0356) compared to mock infected. Next, we tested the levels of IL-1β mRNA in the frontal cortex tissues and observed a significant increase in IL-1β expression level in HIV-positive group (*p* = 0.0174) (Fig. 5E), but not TNF-α (*p* = 0.7487) (Additional file 1, Fig S4C). Moreover, we found that normalized expression level of IL-1β positively correlate with Nrgn-AS level (*r* = 0.33, *p* = 0.032) (Fig. 5F), corroborating with the in vitro findings.

To further test this hypothesis in vitro, we exposed differentiated SH-SY5Y to recombinant IL-1β (1 ng/mL) for 1 h and assessed the levels of Nrgn and Nrgn-AS through RT-qPCR. Results indicate similar dysregulation of the sense-antisense axis in which Nrgn-AS, RP11-677M14.2 levels aberrantly increased by 143-fold, whereas Nrgn-mRNA levels decreased by 1.4-fold (Fig. 5G). This suggests that activation of an intracellular cascade downstream to IL-1β leads to transcriptional activation of Nrgn-AS which in turn downregulates Nrgn mRNA.

### Human brain organoids carrying HIV-1 infected microglia recapitulate the Nrgn-Nrgn-AS dysregulation

**Fig. 6:**
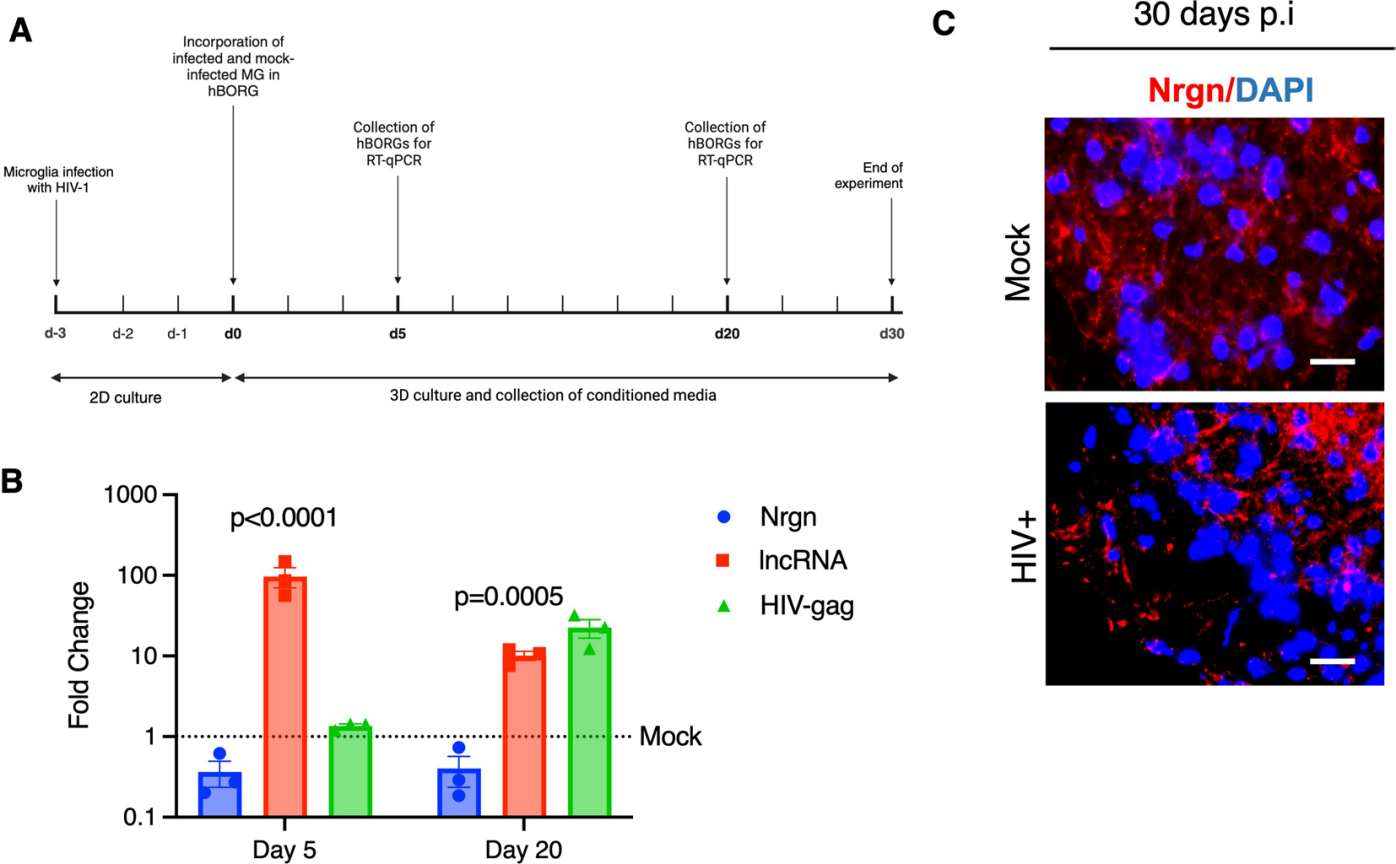
**HIV-1 infection causes Nrgn dysregulation in Brain organoids.** (**A**) Schematic diagram of the experimental design is depicted. Primary adult brain microglia (0.5 × 10^6^ cells) were infected with HIV-1 or mock-infected and were added to brain organoids for overnight. Microglia-embedded organoids were harvested at days 5 and 20 p.i. for RNA extraction, and at days 10 and 30 p.i. for immunostaining. (**B**) Mean of fold change variation in Nrgn, RP11-677M14.2 transcript and HIV-1 Gag expression compared to mock assessed through RT-qPCR (*N* = 3). Dotted lines represent the expression levels of mock-infected brain organoids. (**C**) Immunostaining of infected brain organoid for Nrgn (Cy5, red) and nuclei (blue) compared to mock-infected organoid. DAPI Scale bars show 10 μm, *N* = 3. The images are z-projections of image stacks acquired at ×40 magnification

To study Nrgn-Nrgn-AS dysregulation in a more physiologically relevant model, we leveraged 3D brain organoid technology by incorporating infected microglia to better represent the HIV-1 infected brain microenvironment. Having previously established that the triculture brain organoid system is amenable to HIV-1 infection resulting in increased glial activation and neuroinflammation [26, 39], we used our model to discern the contribution of Nrgn-AS dysregulation to Nrgn levels. As depicted in the schematic (Fig. 6A), we infected primary adult brain microglia with HIV-1 YU2-EGFP (MOI of 0.5) and 3 days post infection, microglia were incorporated into the fully mature brain organoids to generate an immunocompetent brain organoid as described [26]. These organoids were cultured for up to 30 days, harvesting RNA at days 5 and 20 for expression analysis. We found that incorporation of HIV-1 infected microglia caused substantial decrease in Nrgn expression (2.7-fold, *p* = 0.0056), whereas Nrgn-AS expression increased significantly (96-fold, *p* < 0.001) as early as 5 days in infected microglia containing organoids compared to uninfected control organoids (Fig. 6B). The same dysregulation continued up to day 20 p.i. where Nrgn mRNA and proteins levels are continuously suppressed (-2.5-fold, *p* = 0.0005) and Nrgn-AS level is elevated by 10-fold (*p* = 0.005) (Fig. 6B **- C**). In addition, the low expression of HIV-1 Gag at day 5 post incorporation suggests that the rapid Nrgn mRNA dysregulation occurred prior to active viral replication, and it was sustained thereafter corroborating with our hypothesis that the inflammatory environment is driving the Nrgn-Nrgn-AS axis dysregulation. Finally, we assessed the expression of Nrgn protein level by immunohistochemistry at day 30 post-microglia incorporation. As expected, HIV-1 infection led to a decrease of Nrgn immunostaining and accumulation of the remaining protein in the perinuclear region (Fig. 6C).

## Discussion

The events underlying the functional and structural plasticity of neurons are widely thought to be the molecular basis of cognition. Expression level of Neurogranin (Nrgn) highly correlates to neuronal plasticity and cognitive performance in humans [40–42]. We have previously demonstrated that Nrgn is dysregulated in frontal cortex of HIV-associated cognitive disorder (HAND)-positive individuals, triggering the loss of synaptic integrity markers [4]. Herein, we performed an extensive characterization of Nrgn in frontal cortex samples of people with HIV-1, with and without cognitive impairment, compared to age and sex -matched healthy control samples. Nrgn dysregulation at both mRNA and protein levels is evident in the frontal cortex of HIV-1 positive individuals even before the emergence of clinical symptoms of cognitive decline. We showed that Nrgn is dysregulated in individuals with HIV-1 before experiencing dendritic loss and neurodegeneration. This was determined through MAP-2 staining, indicating that the loss of Nrgn could be an early molecular event in the development of HAND. Currently, there is no effective treatment to alleviate or restore cognitive deficits in HAND. Therefore, it is important to elucidate the molecular mechanism(s) underlying Nrgn dysregulation that results in learning and memory impairment. Long non coding RNAs (lncRNAs) are emerging as key players in regulating important cellular functions and a large volume of studies have linked the aberrant lncRNAs expression to a diverse number of human diseases [43]. However, very few lncRNAs are linked to HIV-1 infection and virtually none to the development and/or progression of HIV-1 associated neurocognitive disorder (HAND) [15, 16].

Here we present a novel lncRNA antisense transcript (RP11-677M14.2), named as Nrgn-AS, located in chromosome 11, same as NRGN locus, which function has not been identified. Expression of this lncRNA is negatively correlated with Nrgn mRNA levels in frontal cortex of some HIV-1 positive individuals, suggesting that this lncRNA might be of relevance to Nrgn dysregulation. Thus, based on the in vivo evidence of a discordant regulation of the expression of sense and anti-sense transcripts, which is further supported by *in silico* analyses, we hypothesize that Nrgn-AS functions to suppress Nrgn expression.

Supporting our hypothesis, overexpression of RP11-677M14.2 in SH-SY5Y cells, led to significant decrease in Nrgn mRNA expression. Conversely, we demonstrated that knockdown of RP11-677M14.2 restore Nrgn mRNA expression. Indeed, a previous RNA Seq assessment of the differential expression of miRNAs and lncRNAs has shown decreased expression of RP11-677M14.2 transcript in amygdala samples of schizophrenia patients in comparison with samples from control patients, whereas the expression of Nrgn mRNA was aberrantly higher in schizophrenia samples compared to controls [44]. Results of this study further strengthen our hypothesis that the dysregulation of the RP11-677M14.2 transcript has important biological and functional relevance. We have also demonstrated a reduction in the basal expression levels of some synaptodendritic markers in neurons stably overexpressing RP11-677M14.2 transcript. Although the exact regulatory impact of RP11-677M14.2 transcript on memory and cognition remains elusive, the available evidence generated in this study and others suggests that the Nrgn-Nrgn-AS axis is important for synaptogenesis and CNS homeostasis and their dysregulation could have profound consequences for the brain function. Future whole transcriptome analysis of cells endogenously overexpressing RP11-677M14.2 and cells silencing RP11-677M14.2 expression will clarify how specific is this anti-sense regulatory mechanism.

Homologous genes tend to have similar molecular and biological functions across organisms. However, lncRNAs loci are highly plastic compared to protein-coding genes which makes the identification of sequence-similar homologs a very complex task [45–47]. Although we could not trace any sequence homologs across species, a previous study surveying natural antisense transcripts in developing mouse brain revealed that multiple antisense transcripts occupy the same neighboring genomic locus that encodes for Nrgn [48], which suggests a syntenic transcription pattern of the neurogranin anti-sense strand. Moreover, the same study reported post-transcriptional regulation of Nrgn through overlapping antisense transcripts during cerebral corticogenesis [48]. Interestingly, our results showing discordant expression levels of sense and antisense before and after SH-SY5Y differentiation, suggests that RP11-677M14.2 acts as a temporal regulator of Nrgn as lncRNA is abundant in neuroprogenitor cells but suppressed soon after the neuronal differentiation is initiated. Altogether, these indicate that there is a spatiotemporal expression pattern of the RP11677M14.2 transcript which might be conserved in mice and humans. Future experimental interrogations will be necessary to determine the functional domains of RP11677M14.2, their respective secondary structural elements and how conserved these structures are. In our perspective, these are fundamental questions to better understand how the RP11677M14.2 transcript influences Nrgn expression in physiology and disease.

Our results from RNA HCR- FISH further demonstrated that RP11-677M14.2 transcript is evenly distributed throughout the cells as several other lncRNAs identified recently [18, 49, 50]. Subcellular localization of lncRNAs generally indicates their putative physiological roles. LncRNAs accumulated in the nucleus primarily mediate gene transcriptional repression or activation [51], whereas, lncRNAs accumulated in the cytoplasm interacts directly and/or indirectly with mRNAs to either stabilize the transcript for translation or target it to degradation [52, 53]. Remains unresolved whether the RP11-677M14.2 transcript exerts multiple roles in different cellular compartments as previously shown for other lncRNAs [54]. Regardless, lncRNA transcripts localization is a dynamic process and all lncRNAs are transcribed in the chromatin and therefore presenting nuclear expression. Eventually the transcripts are exported to the cytoplasm, a dynamic that still can be modified upon different stimuli and/or conditions. Our HCR-FISH results also demonstrated that the RP11-677M14.2 transcript do not physically interact with its counterpart Nrgn mRNA, interpreted by the lack of colocalization of both transcripts, thereby excluding posttranscriptional effects such as direct antisense-mediated degradation. Other possibilities include RP11-677M14.2 transcript control of the chromatin state and Nrgn transcription initiation, co-transcriptional effects, and an indirect modulation of Nrgn mRNA stability in the cytoplasm [35]. RNA pull-down assays followed by mass spectrometry and RNAseq are needed to identify potential RP11-677M14.2 molecular partners.

We also demonstrated that RP11-677M14.2 transcript is upregulated in response to treatment with conditioned medium from HIV-1 infected macrophages/microglia. In our experimental setting, we observed that Nrgn mRNA dysregulation was synchronously influenced by RP11-677M14.2 lncRNA expression. While we cannot be certain that the Nrgn dysregulation upon exposure to supernatant of HIV-1 infected glial cells is due only to the up-regulation of RP11-677M14.2 lncRNA expression, these results provide preliminary evidence for a role to RP11-677M14.2 transcript in HIV-1 neuropathogenesis. Importantly, these observations suggest that the inflammatory factors released by infected macrophages may be in part responsible for Nrgn dysregulation through its anti-sense transcript (RP11-677M14.2). Moreover, our work hints at the possibility to restore Nrgn expression by modulating its antisense expression potentially reversing the cognitive impairment or halting the disease progression. Remains to be investigated, whether treatment to silence RP11-677M14.2 transcript would restore Nrgn in the neurons damaged by the inflammatory conditions induced by HIV-1 infection. Ideally, an effective therapeutic agent should preferentially increase Nrgn levels without disturbing physiologically essential basal expression levels [55]. We speculate that the pharmacological rescue of Nrgn through RP11-677M14.2 transcript could provide a great advance addressing neuroinflammation-related disorders as previously proposed for lncRNAs [56]. In this regard, human brain organoids seem to provide an appropriate model to expand our understanding of this lncRNA function and its modulation in the context of brain inflammation. Furthermore, macrophages/microglia conditioned media also contain virus particles that have not been separated or inactivated. Future experiments will tease out the individual contribution of inflammatory factors, virus particles and viral proteins.

Although our study was limited to the brain, investigation of differential expression of RP11-677M14.2 in plasma and/or CSF may also have a utility as a new biomarker for HAND and other neuroinflammatory conditions, as recently demonstrated for other antisense lncRNA [57].

## Conclusions

In this study, we have employed cellular and molecular approaches to investigate the role of RP11-677M14.2 lncRNA in Nrgn dysregulation. We have demonstrated that upregulation of the lncRNA in HAND may be partially determinant to Nrgn loss in neurons with potential implications to the onset and progression of neuropathogenesis. Insights obtained from this study will further advance our understanding of the molecular mechanism (s) underlying HIV-neuropathogenesis and provide additional opportunities to develop therapeutic targets.

### Electronic supplementary material

Below is the link to the electronic supplementary material.

Supplementary Material 1

Supplementary Material 2

Supplementary Material 3

Supplementary Material 4

Supplementary Material 5

## Data Availability

The data used to support the findings of this study are included within the article and its supplementary material. Sequence of RP11-677M14.2 transcript is publicly available, and it was retrieved from Ensembl (https://www.ensembl.org), Transcript ENST00000531241 from gene ENST00000531241.1. Values for all data points in graphs are available from corresponding author, upon reasonable request.

## References

[1] Saylor D, Dickens AM, Sacktor N, Haughey N, Slusher B, Pletnikov M (2016). HIV-associated neurocognitive disorder–pathogenesis and prospects for treatment. Nat Rev Neurol.

[2] Ellis R, Langford D, Masliah E (2007). HIV and antiretroviral therapy in the brain: neuronal injury and repair. Nat Rev Neurosci.

[3] Everall IP, Heaton RK, Marcotte TD, Ellis RJ, McCutchan JA, Atkinson JH (1999). Cortical synaptic density is reduced in mild to moderate human immunodeficiency virus neurocognitive disorder. HNRC Group. HIV Neurobehavioral Research Center. Brain Pathol.

[4] Guha D, Wagner MCE, Ayyavoo V (2018). Human immunodeficiency virus type 1 (HIV-1)-mediated neuroinflammation dysregulates neurogranin and induces synaptodendritic injury. J Neuroinflammation.

[5] Duskova K, Nagilla P, Le H-S, Iyer P, Thalamuthu A, Martinson J (2013). MicroRNA regulation and its effects on cellular transcriptome in human immunodeficiency virus-1 (HIV-1) infected individuals with distinct viral load and CD4 cell counts. BMC Infect Dis.

[6] Yilmaz A, Fuchs D, Price RW, Spudich S, Blennow K, Zetterberg H (2019). Cerebrospinal fluid concentrations of the synaptic marker neurogranin in Neuro-HIV and Other Neurological disorders. Curr HIV/AIDS Rep.

[7] Dykes IM, Emanueli C (2017). Transcriptional and post-transcriptional gene regulation by long non-coding RNA. Genomics Proteom Bioinf.

[8] Engreitz JM, Haines JE, Perez EM, Munson G, Chen J, Kane M (2016). Local regulation of gene expression by lncRNA promoters, transcription and splicing. Nature.

[9] Nojima T, Proudfoot NJ (2022). Author correction: mechanisms of lncRNA biogenesis as revealed by nascent transcriptomics. Nat Rev Mol Cell Biol.

[10] Briggs JA, Wolvetang EJ, Mattick JS, Rinn JL, Barry G (2015). Mechanisms of long non-coding RNAs in mammalian nervous System Development, plasticity, Disease, and evolution. Neuron.

[11] Wang Q, Zhang D, Feng W, Guo Y, Sun X, Zhang M (2022). Long noncoding RNA TSPOAP1 antisense RNA 1 negatively modulates type I IFN signaling to facilitate influenza a virus replication. J Med Virol.

[12] Barichievy S, Naidoo J, Mhlanga MM (2015). Non-coding RNAs and HIV: viral manipulation of host dark matter to shape the cellular environment. Front Genet.

[13] Earls LR, Westmoreland JJ, Zakharenko SS (2014). Non-coding RNA regulation of synaptic plasticity and memory: implications for aging. Ageing Res Rev.

[14] Ojha CR, Rodriguez M, Dever SM, Mukhopadhyay R, El-Hage N (2016). Mammalian microRNA: an important modulator of host-pathogen interactions in human viral infections. J Biomed Sci.

[15] Imam H, Bano AS, Patel P, Holla P, Jameel S (2015). The lncRNA NRON modulates HIV-1 replication in a NFAT-dependent manner and is differentially regulated by early and late viral proteins. Sci Rep.

[16] Zhang Q, Chen C-Y, Yedavalli VSRK, Jeang K-T (2013). NEAT1 long noncoding RNA and paraspeckle bodies modulate HIV-1 posttranscriptional expression. MBio.

[17] Torkzaban B, Natarajaseenivasan K, Mohseni Ahooyi T, Shekarabi M, Amini S, Langford TD (2020). The lncRNA LOC102549805 (U1) modulates neurotoxicity of HIV-1 Tat protein. Cell Death Dis.

[18] Carrieri C, Cimatti L, Biagioli M, Beugnet A, Zucchelli S, Fedele S (2012). Long non-coding antisense RNA controls Uchl1 translation through an embedded SINEB2 repeat. Nature.

[19] Modarresi F, Faghihi MA, Lopez-Toledano MA, Fatemi RP, Magistri M, Brothers SP (2012). Inhibition of natural antisense transcripts in vivo results in gene-specific transcriptional upregulation. Nat Biotechnol.

[20] Salta E, De Strooper B (2017). Noncoding RNAs in neurodegeneration. Nat Rev Neurosci.

[21] Faghihi MA, Wahlestedt C (2009). Regulatory roles of natural antisense transcripts. Nat Rev Mol Cell Biol.

[22] Clark MB, Johnston RL, Inostroza-Ponta M, Fox AH, Fortini E, Moscato P (2012). Genome-wide analysis of long noncoding RNA stability. Genome Res.

[23] Antinori A, Arendt G, Becker JT, Brew BJ, Byrd DA, Cherner M (2007). Updated research nosology for HIV-associated neurocognitive disorders. Neurology.

[24] Guo L, Rezvanian A, Kukreja L, Hoveydai R, Bigio EH, Mesulam M-M (2016). Postmortem Adult Human Microglia Proliferate in culture to high passage and maintain their response to Amyloid-β. J Alzheimers Dis.

[25] Guha D, Nagilla P, Redinger C, Srinivasan A, Schatten GP, Ayyavoo V (2012). Neuronal apoptosis by HIV-1 vpr: contribution of proinflammatory molecular networks from infected target cells. J Neuroinflammation.

[26] Dos Reis RS, Sant S, Ayyavoo V (2023). Three-Dimensional Human Brain organoids to Model HIV-1 neuropathogenesis. Methods Mol Biol.

[27] Gray F, Lescs MC, Keohane C, Paraire F, Marc B, Durigon M (1992). Early brain changes in HIV infection: neuropathological study of 11 HIV seropositive, non-AIDS cases. J Neuropathol Exp Neurol.

[28] Masliah E, Heaton RK, Marcotte TD, Ellis RJ, Wiley CA, Mallory M (1997). Dendritic injury is a pathological substrate for human immunodeficiency virus-related cognitive disorders. HNRC Group. The HIV Neurobehavioral Research Center. Ann Neurol.

[29] Matesic DF, Lin RC (1994). Microtubule-associated protein 2 as an early indicator of ischemia-induced neurodegeneration in the gerbil forebrain. J Neurochem.

[30] Díez-Guerra FJ (2010). Neurogranin, a link between calcium/calmodulin and protein kinase C signaling in synaptic plasticity. IUBMB Life.

[31] Ma L, Bajic VB, Zhang Z (2013). On the classification of long non-coding RNAs. RNA Biol.

[32] Panni S, Lovering RC, Porras P, Orchard S (2020). Non-coding RNA regulatory networks. Biochim Biophys Acta Gene Regul Mech.

[33] Ma H, Hao Y, Dong X, Gong Q, Chen J, Zhang J (2012). Molecular mechanisms and function prediction of long noncoding RNA. ScientificWorldJournal.

[34] Braga EA, Fridman MV, Burdennyy AM, Loginov VI, Dmitriev AA, Pronina IV et al. Various LncRNA mechanisms in Gene Regulation Involving miRNAs or RNA-Binding proteins in Non-small-cell Lung Cancer: Main Signaling pathways and networks. Int J Mol Sci. 2023;24. 10.3390/ijms241713617 PMC10487663 37686426

[35] Pelechano V, Steinmetz LM (2013). Gene regulation by antisense transcription. Nat Rev Genet.

[36] Katuri A, Bryant J, Heredia A, Makar TK (2019). Role of the inflammasomes in HIV-associated neuroinflammation and neurocognitive disorders. Exp Mol Pathol.

[37] Keledjian K, Makar T, Zhang C, Zhang J, Shim B, Davis H et al. Correlation of HIV-Induced Neuroinflammation and Synaptopathy with impairment of learning and memory in mice with HAND. J Clin Med. 2023;12. 10.3390/jcm12165169 PMC10455390 37629211

[38] Hong S, Banks WA (2015). Role of the immune system in HIV-associated neuroinflammation and neurocognitive implications. Brain Behav Immun.

[39] Dos Reis RS, Sant S, Keeney H, Wagner MCE, Ayyavoo V (2020). Modeling HIV-1 neuropathogenesis using three-dimensional human brain organoids (hBORGs) with HIV-1 infected microglia. Sci Rep.

[40] Kubota Y, Putkey JA, Waxham MN (2007). Neurogranin controls the spatiotemporal pattern of postsynaptic Ca2+/CaM signaling. Biophys J.

[41] Pak JH, Huang FL, Li J, Balschun D, Reymann KG, Chiang C (2000). Involvement of neurogranin in the modulation of calcium/calmodulin-dependent protein kinase II, synaptic plasticity, and spatial learning: a study with knockout mice. Proc Natl Acad Sci USA.

[42] Saunders T, Gunn C, Blennow K, Kvartsberg H, Zetterberg H, Shenkin SD (2023). Neurogranin in Alzheimer’s disease and ageing: a human post-mortem study. Neurobiol Dis.

[43] Niland CN, Merry CR, Khalil AM (2012). Emerging roles for long non-coding RNAs in Cancer and Neurological disorders. Front Genet.

[44] Liu Y, Chang X, Hahn C-G, Gur RE, Sleiman PAM, Hakonarson H (2018). Non-coding RNA dysregulation in the amygdala region of schizophrenia patients contributes to the pathogenesis of the disease. Transl Psychiatry.

[45] Derrien T, Johnson R, Bussotti G, Tanzer A, Djebali S, Tilgner H (2012). The GENCODE v7 catalog of human long noncoding RNAs: analysis of their gene structure, evolution, and expression. Genome Res.

[46] Wood EJ, Chin-Inmanu K, Jia H, Lipovich L (2013). Sense-antisense gene pairs: sequence, transcription, and structure are not conserved between human and mouse. Front Genet.

[47] Hezroni H, Koppstein D, Schwartz MG, Avrutin A, Bartel DP, Ulitsky I (2015). Principles of long noncoding RNA evolution derived from direct comparison of transcriptomes in 17 species. Cell Rep.

[48] Ling K-H, Hewitt CA, Beissbarth T, Hyde L, Cheah P-S, Smyth GK (2011). Spatiotemporal regulation of multiple overlapping sense and novel natural antisense transcripts at the Nrgn and Camk2n1 gene loci during mouse cerebral corticogenesis. Cereb Cortex.

[49] Shen Y, Liu S, Fan J, Jin Y, Tian B, Zheng X (2017). Nuclear retention of the lncRNA SNHG1 by doxorubicin attenuates hnRNPC-p53 protein interactions. EMBO Rep.

[50] Munschauer M, Nguyen CT, Sirokman K, Hartigan CR, Hogstrom L, Engreitz JM (2018). Publisher correction: the NORAD lncRNA assembles a topoisomerase complex critical for genome stability. Nature.

[51] Statello L, Guo C-J, Chen L-L, Huarte M (2021). Gene regulation by long non-coding RNAs and its biological functions. Nat Rev Mol Cell Biol.

[52] Rashid F, Shah A, Shan G (2016). Long non-coding RNAs in the cytoplasm. Genomics Proteom Bioinf.

[53] Batista PJ, Chang HY (2013). Long noncoding RNAs: cellular address codes in development and disease. Cell.

[54] Maida Y, Yasukawa M, Furuuchi M, Lassmann T, Possemato R, Okamoto N (2009). An RNA-dependent RNA polymerase formed by TERT and the RMRP RNA. Nature.

[55] Wahlestedt C (2006). Natural antisense and noncoding RNA transcripts as potential drug targets. Drug Discov Today.

[56] Khorkova O, Stahl J, Joji A, Volmar C-H, Wahlestedt C (2023). Amplifying gene expression with RNA-targeted therapeutics. Nat Rev Drug Discov.

[57] Feng L, Liao Y-T, He J-C, Xie C-L, Chen S-Y, Fan H-H (2018). Plasma long non-coding RNA BACE1 as a novel biomarker for diagnosis of Alzheimer disease. BMC Neurol.

