# Supplementary material for: Neuroinflammation driven by human immunodeficiency virus-1 (HIV-1) directs the expression of long noncoding RNA RP11-677M14.2 resulting in dysregulation of neurogranin in vivo and in vitro
Source: J Neuroinflammation. 2024 Apr 24;21:107. doi: 10.1186/s12974-024-03102-x (PMC11043047; doi:10.1186/s12974-024-03102-x)
Supplement: Supplementary file 1 — Supplementary Material 1 [file 12974_2024_3102_MOESM1_ESM.docx]

**Neuroinflammation driven by Human Immunodeficiency Virus-1 (HIV-1) directs the expression of long noncoding RNA RP11-677M14.2 resulting in dysregulation of Neurogranin *in vivo* and *in vitro.***

*Roberta S. dos Reis^1^, Marc C. E. Wagner^1^, Savannah McKenna^1,^, and Velpandi Ayyavoo^1^*^,*^

^1^Department of Infectious Diseases and Microbiology, School of Public Health**,** University of Pittsburgh, Pittsburgh, PA 15260

**SUPPLEMENTARY MATERIALS-ADDITIONAL FILE 1**


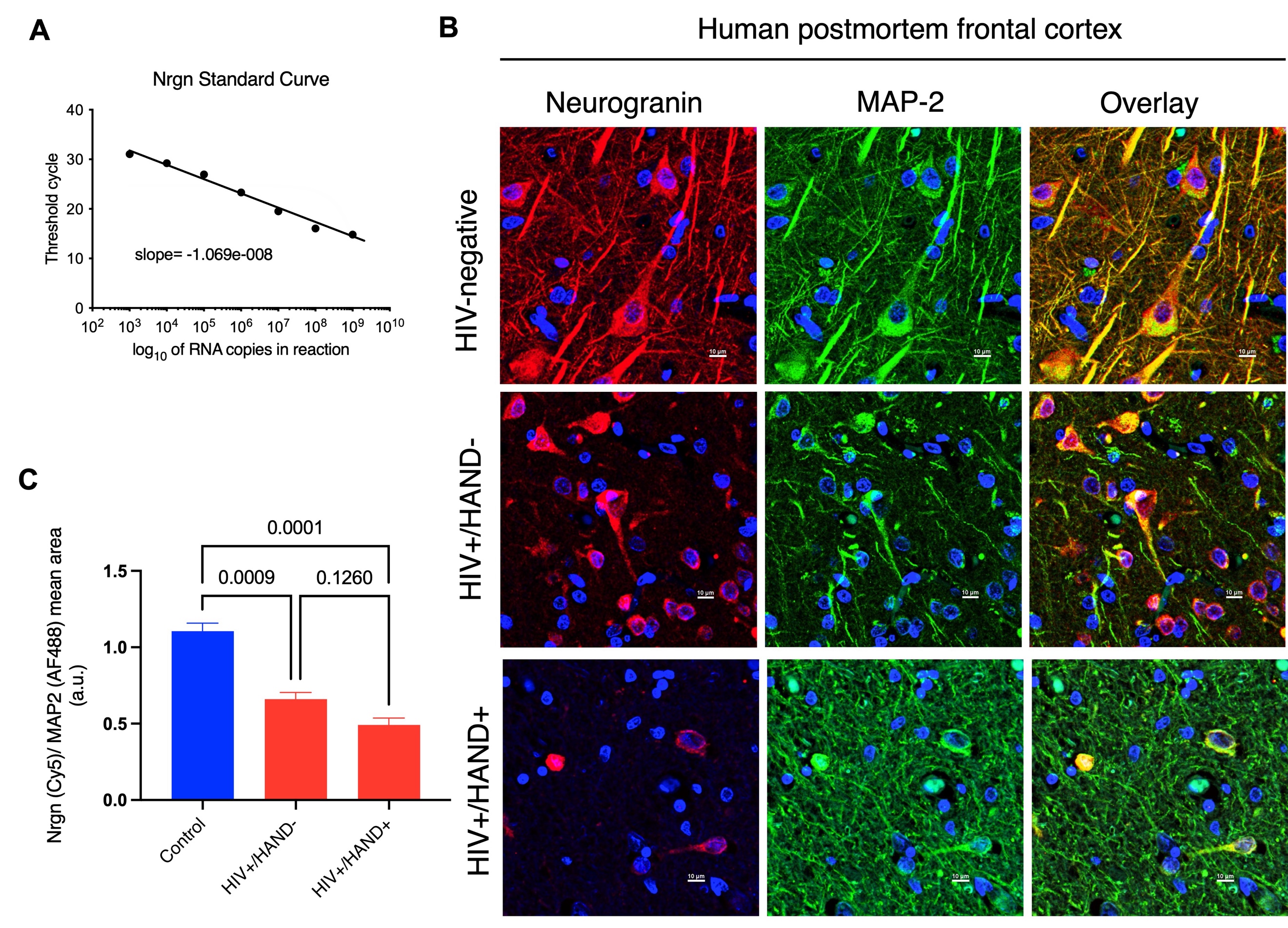


Figure S1. Distribution of Nrgn in frontal cortex and correlation with the dendritic integrity. (A) A standard curve obtained from serial dilutions of full-length Nrgn constructs as template for the qPCR reaction to calculate copy number variation. (B) Representative images of postmortem frontal cortex (FC) neurons co-stained for Nrgn (Cy3, red), MAP-2 (dendritic marker, AF-488, green) and DAPI (blue) in control and people with HIV-1. The images are z-projections of image stacks acquired at ×60 magnification; scale bar is 10 μm. (C) The ratio of the mean intensities of Nrgn and MAP2 staining in HIV-1 positive samples was calculated using the intensity of the colocalization (yellow) normalized to HIV-negative controls (N=4).


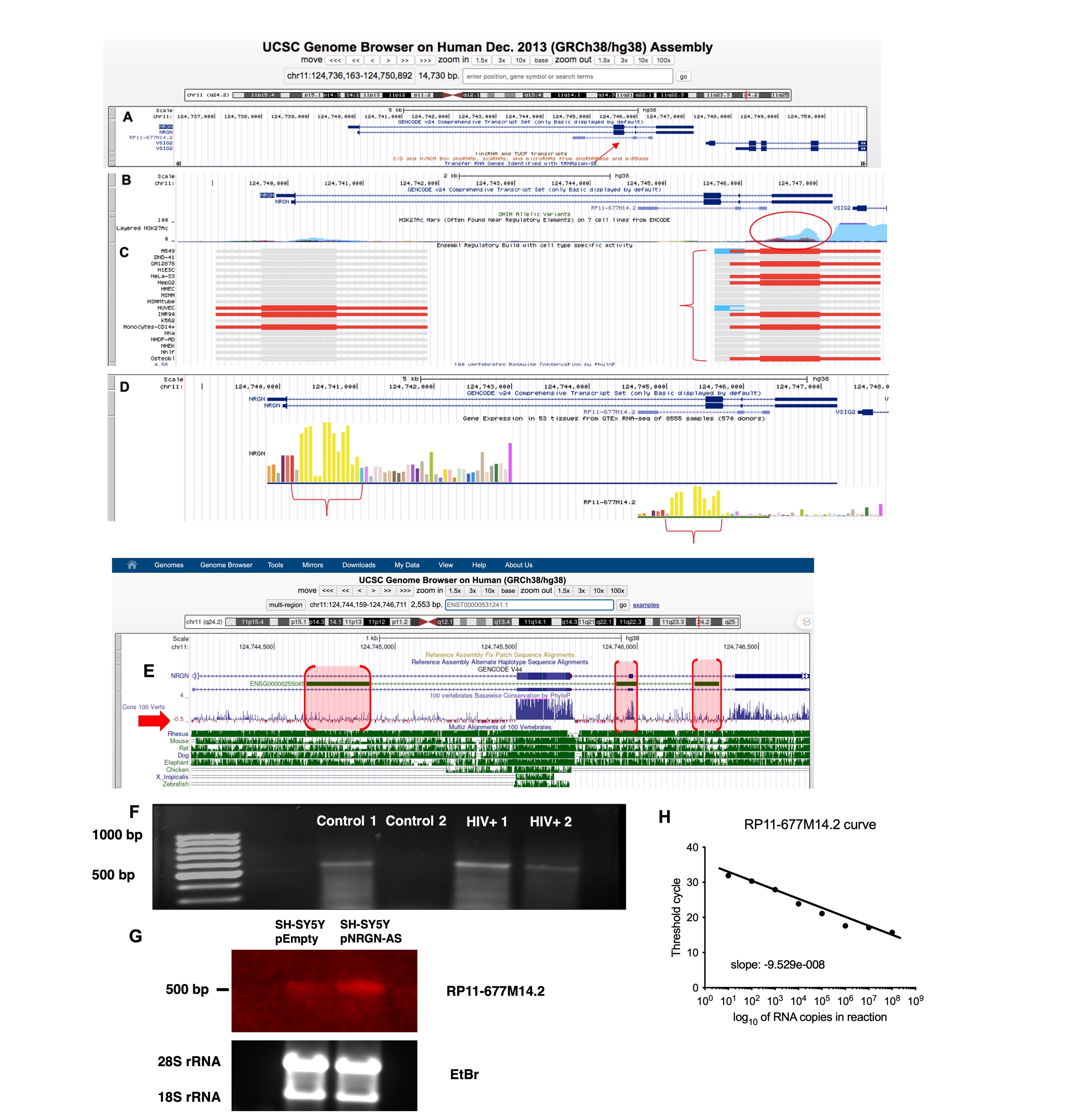


**Figure S2. Identification and characterization of RP11-677M14.2 on Genome Browser.** (**A**) Genome Browser view of human NRGN locus on chromosome 11 is shown. The first two dark blue lines represent the two isoforms of Nrgn mRNA. RP11-677M14.2, shown in light blue, is transcribed from the opposite strand (red arrow). (**B**) Packed ChIP-sequencing data of the NRGN locus for H3K27Ac show active transcription co-aligning with RP11-677M14.2 promoter region (red circle) in several cell lines. (**C**) Expanded ChiP-seq data of the NRGN locus per cell line. (**D**) Nrgn mRNA and RP11-677M14.2 levels measured in 53 different human tissues. Highlighted in yellow are the levels of these two transcripts in different brain regions. (**E**) Full conservation track settings show Vertebrate Multiz Alignment & Conservation and PhiloP conservation of RP11-677M14.2 sequence (red arrow) across 100 species. In the PhyloP plots, blue sites are predicted to be conserved while red sites are predicted to be fast evolving. Based on the multiple alignment, the only nucleotide sequence in the full length RP11-677M14.2 sequence (region shadowed in red) that belongs to a conserved element is in the exon 2. Last accessed in March 2024. (**F**) The expression of RP11-677M14.2 in cDNA synthesized from 1 μg of total RNA from FC brain samples of HIV-1 positive individuals as compared to control brains. (**G**) RP11-677M14.2 probes labeled with AF-647 for Northern blot analysis of undifferentiated SH-SY5Y pEmpty vector and SH-SY5Y pCDNA3.1 Nrgn-AS. The 18S rRNA and 28S rRNA were used as RNA loading controls (**H**) A standard curve obtained from serial dilutions of full-length RP11-677M14.2 construct as template for the qPCR reaction to calculate copy number variation.

**
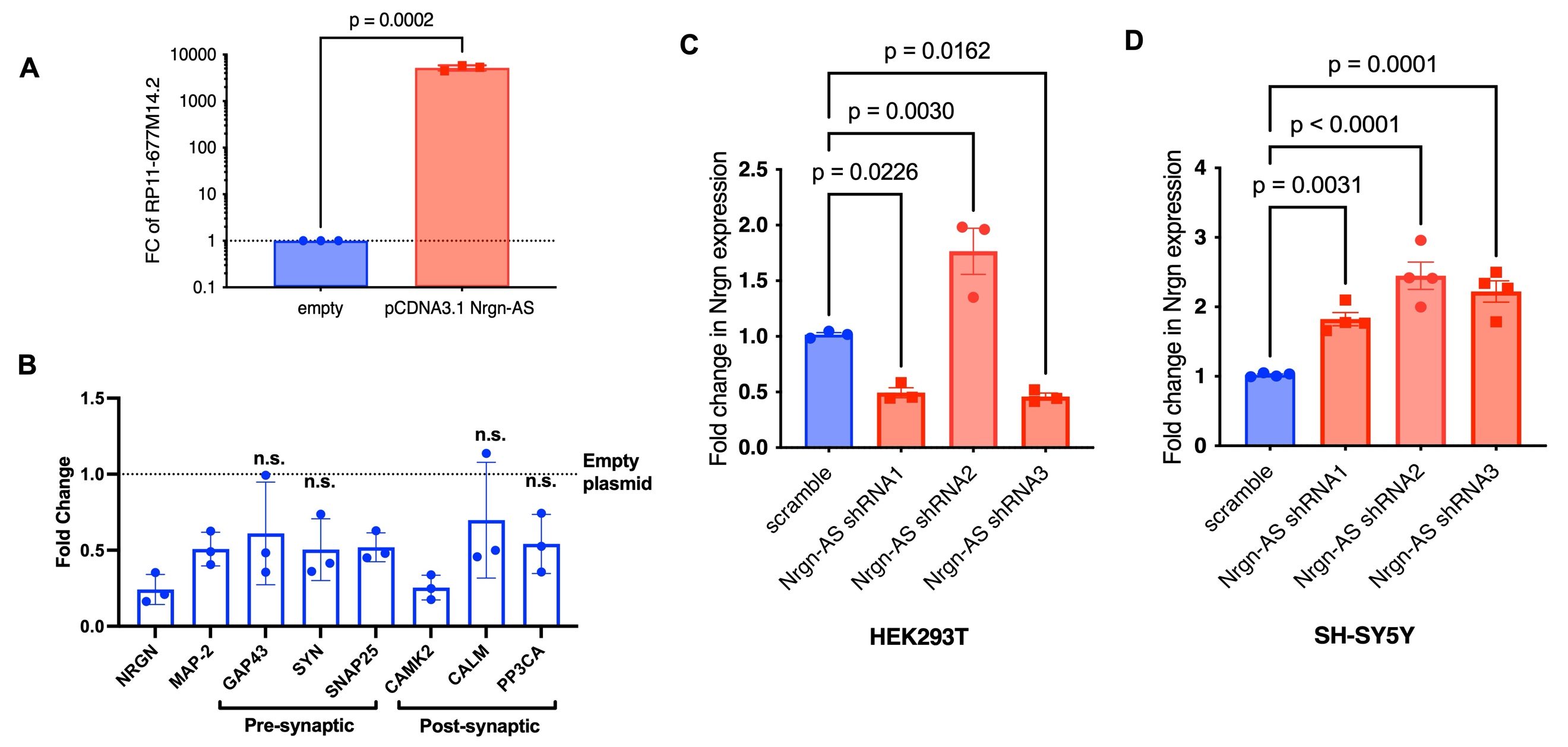
**

**Figure S3.** **Overexpression of RP11-677M14.2 affects synaptodendritic integrity. (A)** Expression level of RP11-677M14.2 of stably transduced SH-SY5Y with pCDNA3.1 Nrgn-AS was assessed by RT-qPCR and compared to empty plasmid. **(B)** Expression levels of synaptodendritic markers were assessed in stably transduced SH-SY5Y: Nrgn (p=0.005641), MAP-2 (dendritic marker, p= 0.016377), GAP43 (pre-synaptic, p= 0.183997), SYN (pre-synaptic, p=0.051), SNAP25 (pre-synaptic, p=0.0128), CAMK2 (post-synaptic, p=0.003930), CALM (post-synaptic, p=0.3029) and PP3CA (post-synaptic, p= 0.054975). (**C-E**) Effect of shRNA1, shRNA2 and shRNA3 targeting RP11-677M14.2 on Nrgn expression in (**C**) HEK293T and (**D**) SH-SY5Y cells assessed by RT-qPCR 72 hrs after transduction (N=4).

**
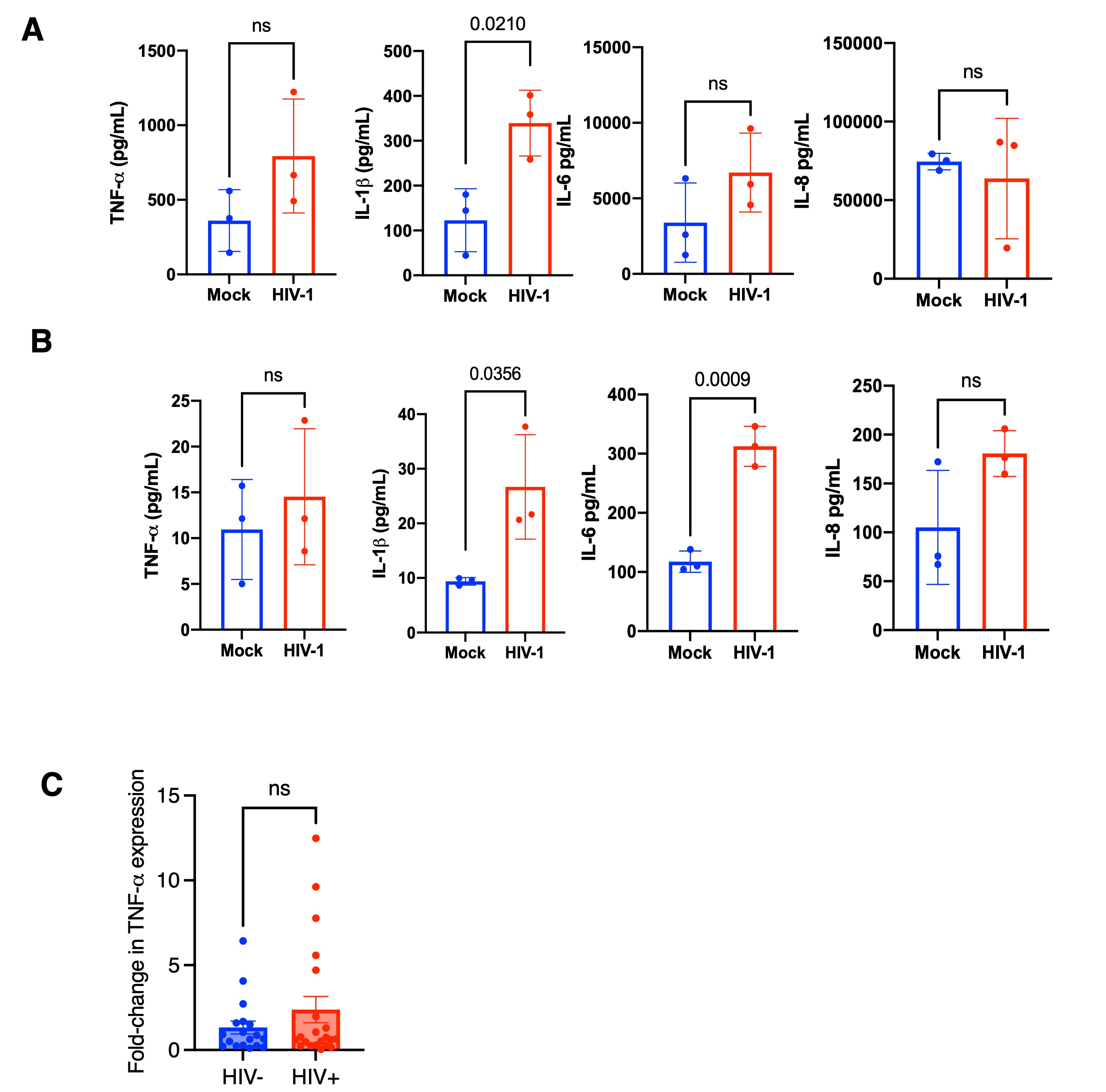
**

**Figure S4. HIV-1 infection of MDM and microglia increase the release of pro-inflammatory cytokines. (A)** Healthy donors-derived macrophages were infected with HIV-1 NLYU2 at MOI=0.5 or mock-infected for 8-12 days. Levels of TNFα, IL-1β, IL-6 and IL-8 in supernatants harvested from mock and HIV-1-infected macrophages were measured by ELISA (N=3 independent experiments from 1 healthy donor). **(B)** Adult primary microglia were infected with HIV-1 NLYU2 at MOI=0.5 or mock-infected and supernatant harvested at day 3 post infection. Levels of TNFα, IL-1β, IL-6 and IL-8 in supernatants were measured by ELISA. **(C)** Relative expression of TNFα in FC brain samples from HIV-1

[1. Saylor D et al. HIV-associated neurocognitive disorder--pathogenesis and prospects for treatment.. *Nat. Rev. Neurol.* 2016;12(4):234–248.](https://sciwheel.com/work/bibliography/4837956)
