# Supplementary material for: Neuroinflammation driven by human immunodeficiency virus-1 (HIV-1) directs the expression of long noncoding RNA RP11-677M14.2 resulting in dysregulation of neurogranin in vivo and in vitro
Source: J Neuroinflammation. 2024 Apr 24;21:107. doi: 10.1186/s12974-024-03102-x (PMC11043047; doi:10.1186/s12974-024-03102-x)
Supplement: Supplementary file 2 — Supplementary Material 2 [file 12974_2024_3102_MOESM2_ESM.docx]

**Neuroinflammation driven by Human Immunodeficiency Virus-1 (HIV-1) directs the expression of long noncoding RNA RP11-677M14.2 resulting in dysregulation of Neurogranin *in vivo* and *in vitro.***

*Roberta S. dos Reis^1^, Marc C. E. Wagner^1^, Savannah McKenna^1,^, and Velpandi Ayyavoo^1^*^,*^

^1^Department of Infectious Diseases and Microbiology, School of Public Health**,** University of Pittsburgh, Pittsburgh, PA 15260

**SUPPLEMENTARY INFORMATION-ADDITIONAL FILE 2**

**Supplementary table 1:** Study cohort characteristics and demographics based on HIV-1 and HAND status.

| Human subject | Repository | Sex | Age (yr) | Race | HIV status | HAND status |
| --- | --- | --- | --- | --- | --- | --- |
| 1 | NNTC | Male | 66 | - | Negative | n/a |
| 2 | NNTC | Male | 64 | - | Negative | n/a |
| 3 | NNTC | Male | 62 | - | Negative | n/a |
| 4 | NNTC | Male | 46 | - | Negative | n/a |
| 5 | NNTC | Male | 57 | - | Negative | n/a |
| 6 | NNTC | Male | 72 | - | Negative | n/a |
| 7 | Neurobiobank | Male | 59 | White | Negative | n/a |
| 8 | Neurobiobank | Male | 59 | Black | Negative | n/a |
| 9 | Neurobiobank | Male | 45 | Black | Negative | n/a |
| 10 | Neurobiobank | Male | 49 | White | Negative | n/a |
| 11 | Neurobiobank | Male | 56 | White | Negative | n/a |
| 12 | Neurobiobank | Male | 51 | White | Negative | n/a |
| 13 | Neurobiobank | Male | 53 | Black | Negative | n/a |
| 14 | Neurobiobank | Male | 56 | White | Negative | n/a |
| 15 | Neurobiobank | Male | 45 | Black | Negative | n/a |
| 16 | Neurobiobank | Male | 50 | Black | Negative | n/a |
| 17 | Neurobiobank | Male | 51 | White | Negative | n/a |
| 18 | Neurobiobank | Male | 52 | Black | Negative | n/a |
| 19 | Neurobiobank | Male | 52 | White | Negative | n/a |
| 20 | Neurobiobank | Male | 53 | White | Negative | n/a |
| 21 | Neurobiobank | Male | 54 | White | Negative | n/a |
| 22 | Neurobiobank | Male | 58 | White | Negative | n/a |
| 23 | MACS | Male | 40 | White | Positive | Negative |
| 24 | MACS | Male | 28 | White | Positive | ANI |
| 25 | MACS | Male | 39 | White | Positive | HAD |
| 26 | MACS | Male | 32 | - | Positive | ANI |
| 27 | MACS | Male | 67 | Black | Positive | ANI |
| 28 | MACS | Male | 40 | White | Positive | MND |
| 29 | MACS | Male | 46 | White | Positive | Negative |
| 30 | MACS | Male | 61 | Black | Positive | Negative |
| 31 | MACS | Male | 39 | White | Positive | Negative |
| 32 | MACS | Male | 54 | White | Positive | ANI |
| 33 | MACS | Male | 38 | White | Positive | HAD |
| 34 | MACS | Male | 52 | White | Positive | MND |
| 35 | MACS | Male | 47 | White | Positive | ANI |
| 36 | MACS | Male | - | - | Positive | HAD |
| 37 | MACS | Male | - | - | Positive | HAD |
| 38 | MACS | Male | - | - | Positive | HAD |
| 39 | MACS | Male | - | - | Positive | HAD |
| 40 | MACS | Male | 46 | Black | Positive | HAD |
| 41 | MACS | Male | - | - | Positive | - |
| 42 | MACS | Male | - | - | Positive | MND |
| 43 | MACS | Male | 47 | White | Positive | HAD |
| 44 | MACS | Male | 49 | White | Positive | - |
| 45 | MACS | Male | 60 | Black | Positive | HAD |
| 46 | MACS | Male | 34 | White | Positive | ANI |
| 47 | MACS | Male | - | - | Positive | MND |
| 48 | MACS | Male | 36 | White | Positive | - |
| 49 | MACS | Male | 45 | White | Positive | ANI |
| 50 | MACS | Male | 45 | White | Positive | Negative |
| 51 | MACS | Male | 52 | Black | Positive | - |
| 52 | MACS | Male | 57 | Black | Positive | HAD |
| 53 | MACS | Male | 49 | White | Positive | HAD |
| 54 | MACS | Male | - | - | Positive | Negative |
| 55 | MACS | Male | 52 | White | Positive | Negative |
| 56 | MACS | Male | 66 | White | Positive | MND |
| 57 | MACS | Male | 34 | White | Positive | HAD |
| 58 | MACS | Male | - | - | Positive | HAD |
| 59 | MACS | Male | 43 | White | Positive | HAD |
| 60 | MACS | Male | 38 | Black | Positive | HAD |
| 61 | MACS | Male | 46 | White | Positive | HAD |
| 62 | MACS | Male | 45 | White | Positive | HAD |
| 63 | MACS | Male | 38 | White | Positive | HAD |
| 64 | MACS | Male | 57 | White | Positive | HAD |
| 65 | MACS | Male | 32 | White | Positive | HAD |
| 66 | NNTC | Male | - | - | Positive | - |
| 67 | NNTC | Male | - | - | Positive | - |
| 68 | NNTC | Male | - | - | Positive | - |
| 69 | NNTC | Male | - | - | Positive | - |
| 70 | NNTC | Male | - | - | Positive | - |
| 71 | MACS | Male | 37 | White | Positive | ANI |

(-) not available; n/a not applicable; ANI asymptomatic neurocognitive impairment; MND mild neurocognitive impairment; HAD HIV-associated dementia.

**Supplementary table 2:** RT-PCR primer sets and probes used in this study.

| mRNA | Description | 5’-3’ sequence | |
| --- | --- | --- | --- |
| Nrgn mRNA1 | Assay ID | Applied Biosystems TaqMan® Gene Expression Assays ID: Hs00183469 |  |
| Nrgn copy number | Assay ID | Applied Biosystems TaqMan® Gene Expression Assays ID: Hs00726070 |  |
| GAP43 | Assay ID | Applied Biosystems TaqMan® Gene Expression Assays ID: Hs00967138 |  |
| MAP2 | Assay ID | Applied Biosystems TaqMan® Gene Expression Assays ID: Hs00258900_m1 |  |
| SYN | Assay ID | Applied Biosystems TaqMan® Gene Expression Assays ID: Hs00300531 |  |
| SNAP25 | Assay ID | Applied Biosystems TaqMan® Gene Expression Assays ID: Hs00938957 |  |
| CAMK2 | Assay ID | Applied Biosystems TaqMan® Gene Expression Assays ID: Hs00947041 |  |
| CALM | Assay ID | Applied Biosystems TaqMan® Gene Expression Assays ID: Hs00237238 |  |
| PP3CA | Assay ID | Applied Biosystems TaqMan® Gene Expression Assays ID: Hs00174223 |  |
| GAPDH | Assay ID | Applied Biosystems TaqMan® Gene Expression Assays ID: Hs00266705 |  |
| Malat-1 | Assay ID | Applied Biosystems TaqMan® Gene Expression Assays ID: Hs00273907 |  |
| RPLP0 | Assay ID | Applied Biosystems TaqMan® Gene Expression Assays ID: Hs00420895 |  |
| SS-Gag | Primer F | TCTCTAGCAGTGGCGCCCGAACA |  |
|  | Primer R | TCTCCTTCTAGCCTCCGCTAGTC |  |
|  | Probe | CGGGAGTACTCACCAGTCGCCGCCCCTCGCCCTCCCG |  |
| RP11-677M14.2  (Nrgn-AS qPCR) | Primer F | GTG TTC ATT ACC TTT GAA AAT GCT CAG |  |
|  | Primer R | TCT CCT ATC TCC TGT GTT TGA GTT CAT C |  |
|  | Probe | AGG GAG GAT GCA GAG GGA |  |
| RP11-677M14.2  (Nrgn-AS RT-PCR) | Primer F | CTT TCA GTA CCA GGA TTC TTT GGG |  |
|  | Primer R | AGG TAC GTA ATA GCT TTA TTT TGG GG |  |
