# Supplementary figures and images for: Neuroinflammation driven by human immunodeficiency virus-1 (HIV-1) directs the expression of long noncoding RNA RP11-677M14.2 resulting in dysregulation of neurogranin in vivo and in vitro
Source: J Neuroinflammation. 2024 Apr 24;21:107. doi: 10.1186/s12974-024-03102-x (PMC11043047; doi:10.1186/s12974-024-03102-x)

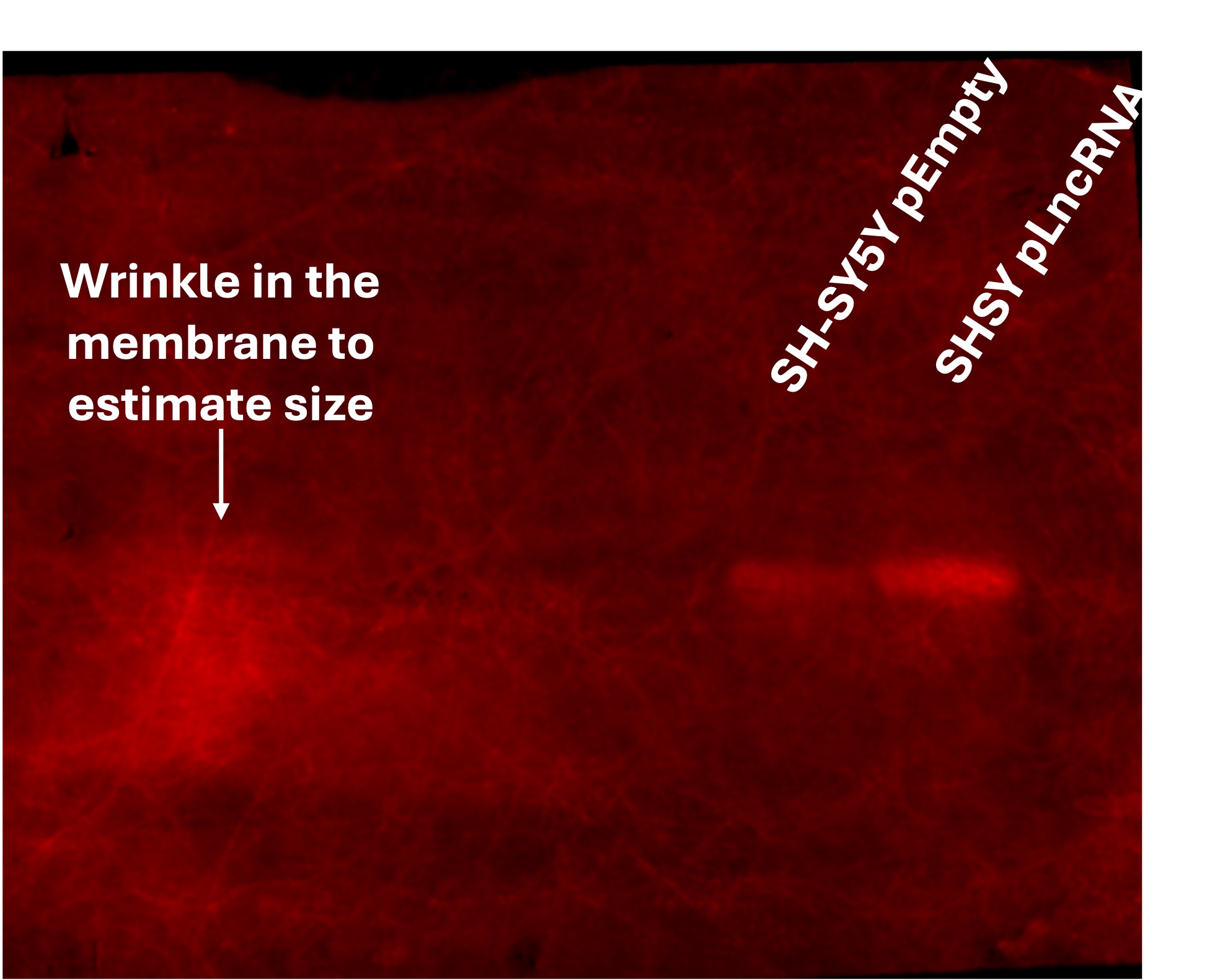

Supplement: Supplementary file 3 — Supplementary Material 3 [file 12974_2024_3102_MOESM3_ESM.jpg]

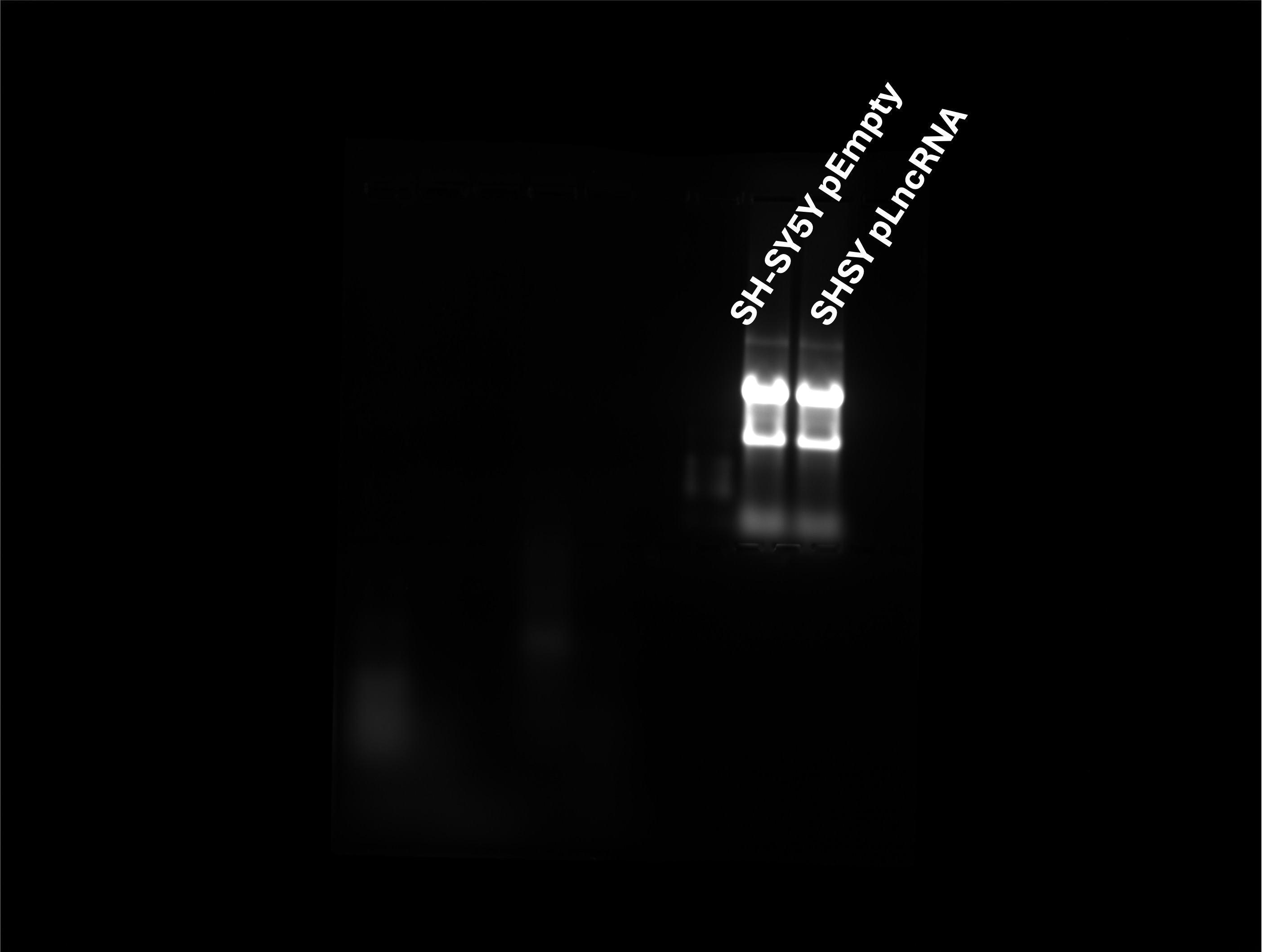

Supplement: Supplementary file 4 — Supplementary Material 4 [file 12974_2024_3102_MOESM4_ESM.jpg]

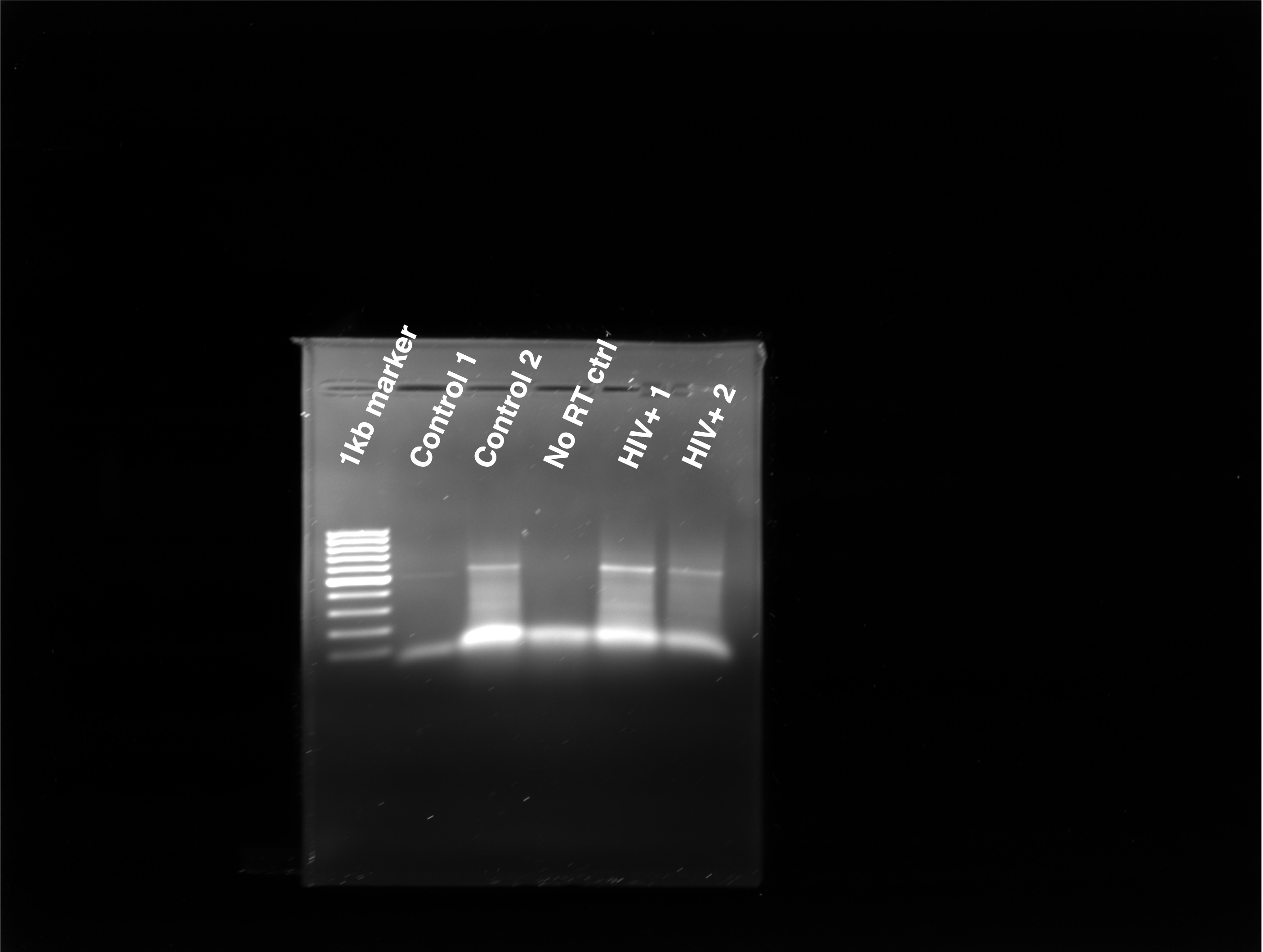

Supplement: Supplementary file 5 — Supplementary Material 5 [file 12974_2024_3102_MOESM5_ESM.jpg]
